# State-of-the-Art Review of Capabilities and Limitations of Polymer and Glass Fibers Used for Fiber-Reinforced Concrete

**DOI:** 10.3390/ma14020409

**Published:** 2021-01-15

**Authors:** Behrouz Shafei, Maziar Kazemian, Michael Dopko, Meysam Najimi

**Affiliations:** 1 Department of Civil, Construction, and Environmental Engineering, Iowa State University, Ames, IA 50011, USA; kazemian@iastate.edu (M.K.); michael.dopko@yahoo.com (M.D.); najimi@iastate.edu (M.N.); 2 Department of Materials Science and Engineering, Iowa State University, Ames, IA 50011, USA

**Keywords:** fiber-reinforced concrete, polymer fibers, glass fibers, micro and macrofibers, fresh and hardened properties

## Abstract

The concrete industry has long been adding discrete fibers to cementitious materials to compensate for their (relatively) low tensile strengths and control possible cracks. Extensive past studies have identified effective strategies to mix and utilize the discrete fibers, but as the fiber material properties advance, so do the properties of the cementitious composites made with them. Thus, it is critical to have a state-of-the-art understanding of not only the effects of individual fiber types on various properties of concrete, but also how those properties are influenced by changing the fiber type. For this purpose, the current study provides a detailed review of the relevant literature pertaining to different fiber types considered for fiber-reinforced concrete (FRC) applications with a focus on their capabilities, limitations, common uses, and most recent advances. To achieve this goal, the main fiber properties that are influential on the characteristics of cementitious composites in the fresh and hardened states are first investigated. The study is then extended to the stability of the identified fibers in alkaline environments and how they bond with cementitious matrices. The effects of fiber type on the workability, pre- and post-peak mechanical properties, shrinkage, and extreme temperature resistance of the FRC are explored as well. In offering holistic comparisons, the outcome of this study provides a comprehensive guide to properly choose and utilize the benefits of fibers in concrete, facilitating an informed design of various FRC products.

## 1. Introduction

Conventional concrete is a brittle material by nature, with a (relatively) weak performance in tension. To alter this characteristic and avoid a sudden brittle failure of concrete structures, reinforcing materials are embedded into the concrete matrix. Since ancient times, people have been putting fibers, such as straws and hairs, in mortars and bricks to improve their tensile properties. These ancient and simple methods of concrete reinforcement have now been transformed into the advanced methods that involve using discontinuous fibers distributed randomly throughout the concrete matrix [1]. The resulting composite material is called fiber-reinforced cementitious composite, even though there are other names for concretes, mortars, or pastes that include fibers within their matrices. The current review study focuses mainly on the cementitious composites that include coarse aggregates, which are commonly referred to as fiber-reinforced concrete (FRC).

The main fiber properties that govern the performance of FRC in the fresh and hardened states include fiber dimensions, elastic modulus, tensile strength, ultimate strain, and bonding and chemical compatibility with the matrix. Considering different fiber materials used in the current practice, the four main categories of metallic, glass, polymer, and natural fibers can be identified. As the name implies, metallic fibers refer to the fibers that are made from metals. The most common type of metallic fibers is steel fibers, but stainless-steel fibers have recently gained a growing attention because of high corrosion resistance. Glass fibers are broadly defined as the fibers that are derived from naturally occurring minerals or rocks. The two general types of glass materials used as fiber reinforcement in cementitious matrices are silica and basalt glass. Polymer fibers are considered as manmade fibers that are neither metallic nor glass fibers. A wide variety of synthetic polymer fibers are deemed suitable for application in FRC, including but not limited to polypropylene (PP), nylon, polyvinyl alcohol (PVA), polyolefin (PO), carbon, polyethylene (PE), polyester (PET), acrylic (PAN), and aramid. Natural fibers are the fibers that occur in nature within the organic tissue of plants. In this study, the main properties of FRC with various polymer and glass fibers are explored in detail, while an investigation of the effects of metallic and natural fibers is deferred to a separate study.

### 1.1. FRC with Micro and Macrofibers

The inclusion of fibers is known to affect various fresh and hardened properties of concrete, while the primary goal of including fibers in concrete is often to prevent (or control) the propagation of cracks. Cracking in concrete is a multiscale process, which starts at the micro-scale when cracks begin to form under applied stresses in the interfacial transition zones (ITZs) between the cement paste and aggregates. Such microcracks spread through the paste until they meet other microcracks, and eventually, grow into a large macrocrack [2]. Once a macrocrack has formed and the crack has widened past the stage of aggregate interlock, the concrete is left with close to no effective tensile load bearing capacity. This mechanism can form anywhere that a high tensile stress is present in the concrete with no reinforcement to prevent the tensile failure as the crack widens. Because of the multiscale progression of cracks in concrete, different sizes of fibers dispersed throughout the concrete matrix are deemed beneficial at different stages of crack growth. It is important to note that if coarse aggregates are taken out of FRC, the fresh and hardened properties of the composite change drastically due to the increased homogeneity of the matrix. The resulting fiber-reinforced mortars are often referred to as high-performance fiber-reinforced cementitious composites and the following micro/macrofiber discussions do not necessarily apply to them.

There are a range of crack-inducing phenomena in concrete, including early-age plastic shrinkage cracks, which can be reduced (or even eliminated) by the inclusion of fibers at low dosages. The inclusion of fibers can also be beneficial for controlling the cracks after hardening. This is highly dependent on not only the fiber type and dosage, but also the dimensions of the fibers added to the cementitious matrix. Microfibers are low diameter, high aspect (length/diameter) ratio fibers that are most often less than 18 mm in length. Microfibers can be effective at arresting microcracks as they extend beyond the ITZ and propagate through the cement paste. This is primarily achieved by bridging the microcracks, facilitating the transfer of tensile stresses. Due to the micro-scale reinforcement action provided by microfibers, it is generally accepted that they most significantly affect the strength properties of FRC prior to full crack formation, as characterized by the stress-strain curve prior to the peak stress. Ultimate strength gains in both compression and tension have been reported for FRC with microfiber [3,4,5], depending on the fiber and matrix properties. Considering that concrete strength increases over time, even low-modulus microfibers can be effective at increasing the strength at an early age. For mature, and especially high-strength concrete, however, pre-crack strength increases are often obtained by using high-strength and high-modulus microfibers. Microfibers generally tend to have a more profound effect on the workability of concrete compared to macrofibers at equal volumetric dosages. This can be associated with the high surface area per unit volume that microfibers typically offer. In order to maintain workability, a sufficient paste volume is needed within the system to coat the additional surface area of the fibers. Alternatively, high dosages of superplasticizers can be utilized. As reflected in the literature, increasing the aspect ratio of the mixed fibers decreases the workability of concrete [6,7,8].

Macrofibers are characterized by their increased lengths (and reduced aspect ratios) compared to microfibers. There is no set standard to define the size boundaries between micro and macrofibers, which creates some overlap in the definitions. However, macrofibers are rarely shorter than 18 mm and generally have diameters larger than 0.1 mm. Macrofibers are effective at bridging the cracks in concrete once they have grown past the micro stage. This is because macrofibers are long enough to provide stress transfer across crack openings when a single crack has formed from the growth of microcracks. If the fiber-matrix bond condition is kept unchanged, the higher the elastic modulus of the macrofiber, the smaller the crack width under the same applied load. This feature relies on the existence of sufficient bond between the fibers and the matrix to develop the strength of the individual fibers and utilize their high stiffness. Besides reported exceptions for the fibers with a high modulus of elasticity [3,9], macrofibers do not significantly influence the strength parameters of concrete prior to crack formation. The effectiveness of macrofibers at bridging cracks depends on the maximum aggregate size as well. In general, for larger aggregates, longer fibers are more effective at improving the post-crack performance, while for smaller aggregates, shorter fibers can be equally (if not more) effective [10]. Due to the fact that microfibers are most effective at improving performance parameters at the early age (for example, by reducing plastic shrinkage cracks) and macrofibers are most effective for post-crack ductility and macrocrack control in FRC, the proper choice of fiber geometry is of the utmost importance for achieving the expected performance, depending on the target application.

### 1.2. Fiber Material Types

In this study, the properties of FRC containing different polymer and glass fibers are investigated with necessary details and comparisons. Table 1 and Table 2 summarize the main characteristics of various polymer and glass fibers, respectively. Many types of polymer fibers have been used in FRC products with various outcomes, mainly due to the diversity of the chemical, physical, and dimensional properties of this category of fibers. The polymer fibers reviewed in this study consist of polypropylene (PP), nylon, polyvinyl alcohol (PVA), polyolefin (PO), carbon, polyethylene (PE), polyester (PET), acrylic (PAN), and aramid. On the other hand, the reviewed glass fibers primarily cover silica and basalt glass fibers. The listed fibers have been subject to adequate research and investigation to warrant their inclusion in the current study.

#### 1.2.1. Polypropylene Fibers

Polypropylene (PP), with a chemical formula of (C_3_H_6_)_n_ [11], is a common type of concrete fiber, owing to its chemical stability in the alkalinity of concrete, wide availability, and low cost. The characteristics and behavior of PP fibers have been extensively explored in the literature [12,13,14,15]. In contrast to steel fibers, PP fibers have a relatively low tensile strength and modulus of elasticity, as listed in Table 1. Although new types of high-tenacity PP fibers have been developed with much higher strength and elastic modulus compared to traditional PP fibers, they are still low in strength and elastic modulus compared to other high-strength concrete fibers. Despite a low strength, the PP fiber is a highly ductile fiber, therefore, it can increase the toughness and impact resistance of concrete, especially at high strains. The PP fiber is one of the most cost-effective concrete fibers available from almost all concrete fiber suppliers.

#### 1.2.2. Nylon Fibers

Nylon is a synthetic fiber with a chemical formula of (C_12_H_22_N_2_O_2_)_n_ that can have a range of strength properties dependent on the base polymer, manufacturing technique, and additives used to make it [16]. Although chemically different, nylon and PP fibers often deliver similar benefits when used in FRC because, in general, they have similar fiber-matrix bond strengths, tensile strengths, and elastic moduli. Nylon fibers are, however, more expensive than PP fibers. Recent interest in recycled nylon fibers is expected to help decrease the cost of this type of fiber. Nylon fibers can be readily obtained from most concrete fiber suppliers.

#### 1.2.3. Polyvinyl Alcohol Fibers

Polyvinyl alcohol (PVA), with a chemical formula of (C_2_H_4_O)_n_, is a relatively high-strength synthetic fiber originally developed for the replacement of asbestos in asbestos cement [17,18]. The use of PVA fibers has been expanded to FRC applications, owing to their satisfactory mechanical properties and ability to bond chemically with the cement matrix. However, PVA fibers are less common in practice since they are more expensive than most other concrete fibers. Although they are not widely available in the concrete fiber market, they can still be purchased from select suppliers.

#### 1.2.4. Polyolefin Fibers

Polyolefin (PO) is a polymer fiber formed by the polymerization of olefin monomer units (C_n_H_2n_) that encompass polypropylene and polyethylene as subgroups [19]. For the purpose of this study, PO fibers are discussed separately due to their distinctions from other polymeric fibers in the FRC literature. PO concrete fibers share similar properties with high-tenacity PP fibers, as shown in Table 1. Because of the similarities between PO and PP fibers, characterized by low tensile strength, low elastic modulus, and high ultimate strain, the performance of FRC products made with these two fibers tends to be similar. It is also common for blended PP/PO copolymer resins to be manufactured into concrete macrofibers. PO fibers are provided by most concrete fiber suppliers, and they have a relatively low price.

#### 1.2.5. Carbon Fibers

Carbon fiber has historically been one of the most popular types of fiber for reinforcing brittle matrix composites to improve their tensile properties [20]. The effectiveness of carbon fiber reinforcement in other types of matrices has sparked interest in using carbon fibers in FRC. Carbon fibers can have a wide range of mechanical properties, depending on the materials used to make the fibers. For example, polyacrylonitrile (PAN)-based carbon fibers have a very high tensile strength and elastic modulus (up to the twice of those of steel fibers), while pitch carbon fibers that are made from petroleum and coal tar pitch have a relatively low tensile strength and elastic modulus. Pitch carbon fibers often exhibit a wide range of tensile strengths and elastic moduli, depending on the nature of the pitch used to make them [8]. The properties of the fibers can vary considerably depending on the manufacturing process as well. Both types of carbon fiber are made from varying degrees of heat treatment, stretching, and oxidation [18]. Carbon fibers are expensive compared to other fiber choices, which limit their use in civil infrastructure applications.

#### 1.2.6. Polyethylene Fibers

Polyethylene (PE) fiber, with a chemical formula of (C_2_H_4_)_n_, can be produced with a wide range of mechanical properties. In the past, PE fibers were being characterized by low strength and elastic modulus, similar to PP and PO fibers. However, the development of ultra-high density PE has greatly increased the strength and stiffness of this type of fiber. From the performance perspective, it can be generally stated that the higher the fiber density and molecular weight, the higher the strength and stiffness potential. These fiber properties depend on the degree of molecular alignment achieved by advanced production processes, involving heat pressure and catalysts [21]. High-strength polyethylene (HSPE) is a type of PE fiber made from gel-spinning ultra-high molecular weight PE. The tensile strength and modulus of elasticity of HSPE are higher than those of other polymeric fibers, as shown in Table 1. HSPE is a high-performance product, thus, it is expensive to buy directly from the manufacturer. However, waste HSPE fibers can be obtained from third-party distributors for a low price.

#### 1.2.7. Polyester Fibers

Polyester fibers generally fall under two categories, i.e., polyethylene terephthalate (PET) and poly(1,4-cyclohexylene dimethylene terephthalate) (PCDT). The PET and PCDT fibers are made using different processes and have different chemical and mechanical properties. PET fibers often have a higher strength and stiffness than PCDT fibers, which are characterized as more ductile. With reference to use as concrete fibers, contrary to PCDT fibers, PET fibers have been subject to extensive research, mostly as fibers recycled from consumer products. Henceforth, the polyester fibers refer to the PET variety. It must be noted that, although PE and PET share the polyethylene name, they are chemically different, as PET is a polyester, not a type of polyethylene.

#### 1.2.8. Acrylic Fibers

Acrylic is a polymer that contains at least 85% acrylonitrile by weight [22], with a chemical formula of (C_3_H_3_N)_n_. The name “acrylic” is short form for, and essentially interchangeable with, polyacrylonitrile (PAN). As previously mentioned, PAN fiber is also the precursor material used to manufacture PAN-based carbon fiber. Acrylic fibers with a high tensile strength and elastic modulus were employed in the form of small-diameter short-cut fibers to replace carcinogenic asbestos [16]. As listed in Table 1, there are a wide range of strength and stiffness parameters that PAN fibers can possess. Due to this variation, the properties of FRC with this type of fiber can also vary substantially. The research pertaining to the performance of PAN fibers in cementitious composites containing coarse aggregates is rather limited, however, there is more evidence in the literature for acrylic fibers in pastes and mortars, likely due to the fact that PAN fibers are predominantly micro in form. Despite a relatively wide availability, acrylic microfibers have a higher price compared to other low-strength synthetic fibers.

#### 1.2.9. Aramid Fibers

Aromatic polyamide is a polymer in which at least 85% of the amide group is bound directly to two aromatic rings [23]. Known in short form as aramid, this fiber has many high-performance applications, owing to its high strength and elastic modulus relative to most other synthetic fibers. Aramid fibers are 2.5 times stronger than silica glass fibers and 5 times stronger than steel fibers per unit weight [16]. These unique qualities have drawn attention to aramid fibers for applications as reinforcement in cementitious matrices. The two most common types of aramid fibers are marketed under the trade names Kevlar and Technora. These two fibers possess different properties, mainly due to differences in their production methods. Kevlar is produced by dry and wet spinning of a sulfuric acid solution of aromatic polyamide, while Technora fiber production does not utilize acid spinning [24]. Aramid fibers are expensive, and they can be hardly found in the concrete fiber market.

#### 1.2.10. Glass Fibers

In this study, glass fibers refer to the fibers that are derived from naturally occurring minerals or rocks, consisting of (SiO_2_)_n_ monomers. Glass fibers are manufactured by extruding melted parent material into a filament form. During the extrusion process, the filaments are coated with a material called sizing, which equips the fibers with the desired surface texture and interfacial properties for the matrix within which they will be used. With regards to glass fibers used in concrete, individual sizing-coated glass filaments are typically gathered into the strands of around 200 filaments and cut to desired lengths. Depending on the production process and intended use, glass strands can be made to disperse back into their filament (microfiber) form when in contact with water (water dispersible) or they can be manufactured to stay in an integral strand (macrofiber) form. A new type of macro glass fiber has been recently developed by impregnating glass strands with an alkali-resistant polymer resin. This type of resin-impregnated fiber follows the same concept as glass fiber-reinforced polymer (GFRP) rebar, only on a smaller scale.

The two main types of glass fibers that have been frequently used in practice as reinforcement in cementitious composites include silica glass and basalt glass. Due to the chemical similarity of their parent materials, the final fiber products are also chemically similar. Basalt and silica glass fibers contain high amounts of silicon dioxide (typically 40% to 70%), depending on the composition of the parent material. The main difference between basalt and silica glass fibers is that basalt glass fibers tend to have significant levels of iron, potassium, magnesium, and sodium oxides, while silica glass fibers typically have low dosages of the mentioned oxides, but they can contain a significant dosage of boron oxides [25]. Although their production methods are similar overall, the production of silica glass fibers often involves the use of additives to improve the physical properties of this type of fiber. Basalt glass fiber production, however, does not require additives, resulting in less consistent fiber properties in the finished product. Furthermore, basalt fiber production is usually a simpler process, making basalt glass fibers less expensive than silica glass fibers [26]. Table 2 summarizes the main properties of silica and basalt glass fibers, although these properties remain highly dependent on the fiber’s parent material and manufacturing process.

#### 1.2.11. Silica Glass Fibers

The first type of glass fibers used for concrete reinforcement was E (or electrical grade) glass. The E glass was originally developed for use in electrical applications. The material was found to have satisfactory mechanical properties and was then tested for use as fiber reinforcement in polymer matrices and eventually cementitious matrices. Due to the degradation of glass fibers in concrete, however, alkali-resistant (AR) glass fibers were later developed. AR glass fibers have a relatively high tensile strength and elastic modulus compared to most polymer fibers. The most common application of AR glass concrete fibers is thin sheet components for exterior façade panels [16]. Such panels are typically made from pastes or mortars that include high fiber volumes [27]. Due to the use of AR glass fibers primarily as thin sheet components, AR glass textile concrete has been developed, in which two- or three-dimensional woven glass fabrics are cast into mortars using a lay-up technique to produce several layers of aligned glass fiber reinforcement [28]. AR glass fibers, which are expected to maintain a set of minimum requirements based on ASTM C1666 [29], are very common and widely available in the concrete market. In general, the cost of AR glass fibers is relatively low.

#### 1.2.12. Basalt Glass Fibers

In recent years, basalt glass fibers have received growing attention in the fiber concrete industry. Basalt glass fibers typically have a higher elastic modulus and tensile strength than silica glass fibers, as listed in Table 2. They are anticipated to gain further popularity in the concrete market as their production increases and unit cost drops. The recent popularity of basalt concrete fibers has provided abundant research in the literature on their contributions to the properties of fresh and hardened FRC. Basalt microfibers are available from a number of concrete fiber suppliers. Basalt fiber-reinforced polymer (BFRP) macrofibers are somewhat less popular in the market to date; however, they are available from select suppliers.

### 1.3. Scope and Organization

The state-of-the-art review presented through this study aims to provide a holistic guide on the capabilities, limitations, and potential applications of different types of polymer and glass fibers used in concrete categorized by their effects on various fresh and hardened properties of FRC. Building on the past efforts [22,30,31,32,33,34,35,36,37,38], a significant number of relevant studies have been reviewed and their main observations and conclusions have been synthesized. This has led to detailed comparisons with the ultimate goal of shedding light on the effects of fiber type on the stability and bond (Section 2), workability (Section 3), pre-peak mechanical properties (Section 4), post-peak mechanical properties (Section 5), shrinkage (Section 6), and extreme temperature resistance (Section 7) of FRC products from both scientific and practical perspectives. Finally, a detailed comparison of the reviewed fibers has been synthesized to further elaborate on their capabilities and limitations (Section 8). It is important to note that the reported outcomes are dependent on not only the fiber material and volume dosage, but also the fiber dimension and matrix composition used in the individual study, which should be considered when comparing experimental test results across the studies and fiber types. This review study is expected to benefit a wide spectrum of researchers and engineers in the concrete industry to properly choose the most appropriate concrete fibers, based on their target applications and desired performance characteristics.

## 2. Stability and Bond

Concrete’s matrix is known to be corrosive to certain materials due to its very high pH value, originating from highly alkaline hydration products. Therefore, it is important to ensure the chemical stability of any fibers added to FRC before investigating their properties and suggesting possible applications. Stability of fibers refers to their ability to withstand the concrete environment during their expected service life without experiencing material degradation or dimensional alteration, hence, maintaining their efficiency. In particular, when fibers undergo deterioration, they can lose a portion of their cross-section, which adversely affects their load-carrying capacity, diminishing the advantages of FRC over plain concrete.

As for bond characteristics, the bond between a fiber and its surrounding concrete is quantified as the force needed for the fiber to either be pulled out of the concrete matrix or experience rupture. When a bulk of discontinuous fibers are added to concrete, a combination of the aforementioned failure modes is expected under external loads, based on the fiber-matrix bond, which is influenced by the concrete’s properties and fiber’s characteristics. As stated in Banthia [39], fiber properties (e.g., type, shape, length, and coating), matrix characteristics (e.g., water-to-cement ratio, aggregate size, and admixtures), and environmental and loading conditions contribute to the pull-out behavior of a fiber in concrete. The fiber-matrix bond can be purely mechanical or a combination of mechanical and chemical. Fibers made of chemically inert materials remain unreacted in concrete, thus, the only mechanism to resist the load applied to them is the friction between the individual fibers and their surrounding concrete matrix. On the other hand, when a reaction forms between the fibers and the concrete matrix, the chemical bond helps with the friction resistance, delivering a combination of mechanical and chemical bonds. The fiber-matrix bond characteristics are particularly important in FRC products because they directly affect both pre-peak and post-peak mechanical properties.

### 2.1. Polypropylene Fibers

**Figure 1:**
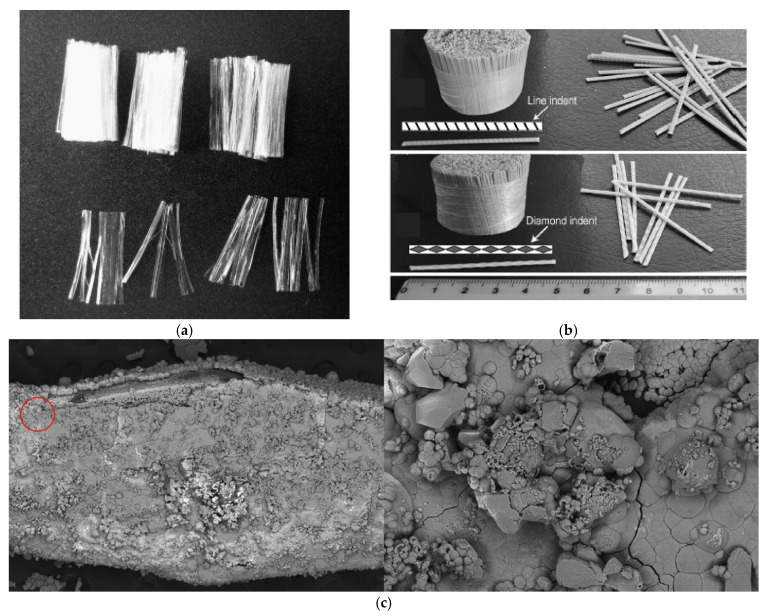
(**a**) Fibrillated PP fibers [43], (**b**) surface-indented PP fibers [42], and (**c**) pre-treated PP fibers with microbially-induced calcite precipitation [44].

The hydrophobic nature of PP fibers often results in an overall weak fiber-matrix bond, leading to a mode of failure governed by the fiber pull-out under external loads. However, if the concrete matrix’s strength is increased sufficiently, or an appropriate mechanical anchorage is provided to the fibers (with geometric modifications), the mode of failure can change to the rupture of individual fibers, utilizing their full capacity. Cifuentes et al. [40] confirmed this assessment by reporting that PP fibers fail due to pull-out in low- and normal-strength concrete, while they failed because of a rupture in high-strength concrete. In order to increase the fiber-matrix bond strength, the fibrillated PP fiber’s bond can be improved by splitting a PP fiber into fibrillated bundles during the mixing process (Figure 1a). The monofilament PP fibers, on the other hand, can have their bond strengths improved by shape variations. Oh et al. [41] tested straight, crimped, hooked, button end, twisted, sinusoidal, and partially-sinusoidal shape synthetic macrofibers for their bond strengths. The study concluded that the crimped and sinusoidal shape monofilament PP fibers exhibit the highest improvement in bond properties, compared to straight monofilament PP fibers. Another common way to increase the fiber-matrix bond strength of monofilament PP fibers is by twisting the straight fibers along their longitudinal axis, or indenting their surfaces (Figure 1b). Yin et al. [42] indicated that diamond surface indentations are more effective than line indentations in increasing the bond of macro PP fibers.

Chemical pre-treatments can also be adopted to increase the fiber-matrix bond for PP fibers. López-Buendía et al. [45] used alkaline surface treatment and found that the adhesion of macro PP fibers to the concrete matrix increases, as a result of longitudinal roughness. In addition, the cited study showed how a chemical adhesion between the individual fibers and the concrete matrix contributes to increasing the flexural strength of FRC products. In a study performed by Hao et al. [44], the microbially-induced calcite precipitation pre-treatment method was investigated. The outcome showed the success of this method in improving the bond between the treated PP fiber and the mortar matrix. This was explained by noting that the calcium carbonate produced as a result of microbial activities increases the surface roughness of the macro PP fiber, as depicted in Figure 1c. Further to the aforementioned methods, high-tenacity PP fibers can be utilized to develop sufficient bond, providing significant post-crack residual strength and toughness. In a recent development, a new type of PP fiber has been produced with the ability to chemically bond with the concrete matrix. When this new type of macro PP fiber was compared to a traditional type of macro PP fiber, both in monofilament form, the fiber pull-out capacity was found to improve by more than 30% [46].

### 2.2. Nylon Fibers

The pull-out behavior of nylon fibers from the concrete matrix is known to be very similar to PP fibers [47]. However, the amide group (–CO–NH–) in nylon fibers forms a reaction with water, which absorbs moisture into the fibers and causes them to swell [48]. By examining the surface of the nylon fibers during pull-out tests, it was noted that the pull-out capacity increases during the loading process because the concrete matrix scars the outside surface of the nylon fiber, effectively increasing the friction between the fiber and the concrete matrix. A similar observation was also made for the micro PP fibers [47]. According to Yap et al. [49], nylon fibers outperformed fibrillated PP fibers in compressive strength tests, due to the hydrolysis of the nylon’s amide group and the subsequent swelling of the fibers, which increased the bond between the nylon fibers and the surrounding concrete. The overall bond behavior of nylon fibers was documented by Khan and Ali [50]. In the cited study, when 50 mm long nylon fibers were tested in a normal-strength concrete under flexural loads, about 70% of the nylon fibers were found to fail due to pull-out, while the remaining 30% failed because of rupture.

### 2.3. Polyvinyl Alcohol Fibers

**Figure 2:**
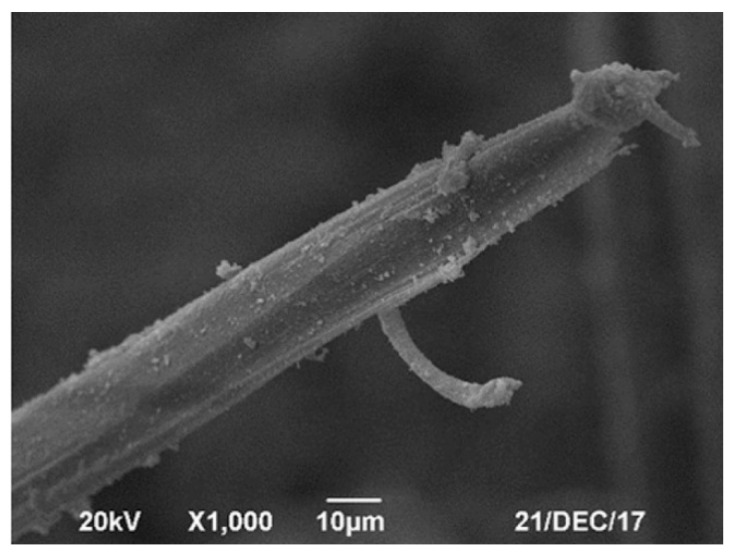
A single PVA fiber after being pulled-out from a cementitious matrix [55].

PVA fibers are characterized as hydrophilic, have a non-circular cross section, and form hydrogen bonds with the concrete matrix. These characteristics give PVA fibers the ability to form a strong bond in FRC applications [22], which is estimated to be eight times higher than the PP fiber’s bond strength [51]. Although PVA fibers are hydrophilic, they have a very low water absorption. PVA fibers are also very compatible with the chemical environment of the concrete matrix, retaining nearly their entire strength after accelerated aging tests equivalent to 100 years [52]. Despite an excellent resistance to acidic and alkaline environments, Roque et al. [53] reported that PVA fibers can show degradation in seawater environments, especially after repeated wetting and drying cycles. It has been indicated in several studies that PVA fibers form both chemical and mechanical bond with the concrete matrix. Through scanning electron microscope (SEM) investigations, Zhao and He [54] revealed the precipitation of the C–S–H gel on PVA fibers. Furthermore, Li et al. [55] showed that the pulled-out PVA fibers undergo a notable diameter loss, as shown in Figure 2, which reflects the strong bond between the PVA fiber and the surrounding matrix. Due to the ability of PVA fibers to chemically bond with the concrete matrix, there is no need to alter the geometric shape of this type of fiber. Thus, PVA fibers are often manufactured in a monofilament form for both macro and micro sizes. PVA fibers tend to fail by rupture rather than pull-out much faster than other fiber types. This has been attributed to a slip-hardening response, originating from strong fiber-matrix bond properties [56,57]. Additionally, it has been reported that the response of PVA fibers shifts from ductile to brittle, as the fiber-matrix bond increases over time. The fiber failure mode can also shift from pull-out to rupture, depending on concrete matrix properties [58].

### 2.4. Polyolefin Fibers

PO fibers are very compatible with the concrete matrix and do not degrade in the concrete environment. The PO fiber-matrix bond is mechanical in nature [59]. Depending on the manufacturing technique, macro PO fibers can be made with surface indentations to enhance their mechanical bond properties [18]. It has been suggested that, since PO fibers have a low superficial hardness, their mechanical bond can be increased as a result of micro-scale surface imperfections that form because of damage to the fibers at the time of mixing. As expected, the bond properties between the PO fibers and the concrete matrix improve with the progress of cement hydration [60]. In particular, with SEM investigations, Han et al. [61] found silica fume helpful in improving the bond between the PO fibers and the concrete matrix. Relatively low-modulus PO fibers were observed to be most effective, along with silica fume, when 25 mm fibers were used in place of 50 mm fibers for improving the mixture’s strength and ductility characteristics [61].

### 2.5. Carbon Fibers

Carbon fibers are chemically inert, and as a result, do not undergo strength deterioration in the concrete environment [18,62,63,64]. Therefore, carbon fibers can only form mechanical bonds with the concrete matrix. Fibers with a high modulus of elasticity, such as carbon fibers, tend to pull out rather than rupture under the external loads applied to FRC. This, however, also depends on the matrix strength and the fiber dimensions, as well as the contact surface area between the fibers and the concrete matrix. Pitch carbon fibers in mortar were found to have sufficient strength to fail by pull-out, unless latex is used to enhance the fiber-matrix bond, in which case the failure mode can shift to fiber rupture [65].

### 2.6. Polyethylene Fibers

High-strength polyethylene (HSPE) fibers are chemically inert, providing high stability and degradation resistance in the concrete environment, in addition to high resistance against acids and seawater. Recycled PE fibers also adequately withstand the alkalinity of the concrete environment [66]. The HSPE fibers have a low coefficient of friction, causing them to form a weak bond with their surrounding matrix [22,67]. The bond strength of the HSPE fibers, however, can be improved by surface treatments. Wu and Li [68] studied such treatments and reported that the fibers can form a bond with the concrete matrix up to a 1.0 MPa strength if a surface finish is applied to them to increase their friction coefficient. Additionally, it was found that plasma treatment of the fibers can considerably increase the fiber-matrix bond strength [68]. In a separate study, He et al. [69] showed that coating the HSPE fibers with carbon can increase their frictional bond strength by more than 20%. As stated by Pešic et al. [66], recycled high-density PE fibers often fail due to the pull-out caused by mechanical friction, while they undergo high elongations before being pulled out of the concrete matrix. Through SEM investigations, the cited study confirmed that those fibers do not form a chemical bond with the concrete matrix.

### 2.7. Polyester Fibers

**Figure 3:**
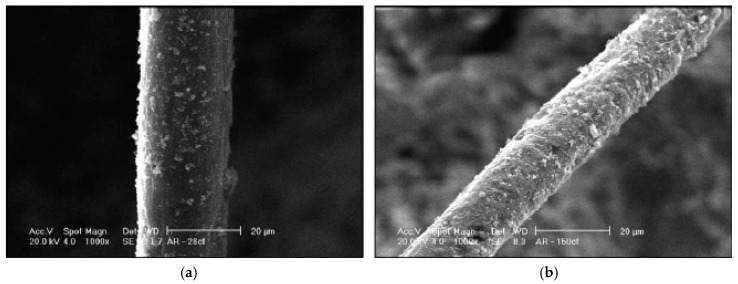
SEM images of recycled PET fibers after (**a**) 42 days and (**b**) 164 days of exposure in the cement mortar [73].

Despite an overall promise, the main concern with the use of polyester fibers in cementitious composites is the uncertainty with regards to their stability in the highly alkaline environment of concrete. Most of the available studies have reported some level of degradation after a prolonged exposure of this type of fibers to extreme environments. Kim et al. [70] studied recycled PET FRC for strength retention after exposure to alkaline and acidic solutions. It was reported that an exposure to such solutions not only reduces the strength of PET fibers, but also significantly deteriorates the physical and mechanical properties of the entire concrete matrix. These observations were further supported by Fraternali et al. [71], which reported that, after 12 months in an aggressive seawater curing environment, toughness of recycled PET FRC dropped by more than half. In a separate effort, Rostami et al. [72] confirmed the past findings and reported a tensile strength loss over time. Additionally, Silva et al. [73] showed that PET FRC can suffer from the loss of toughness during the expected service life. The cited study used SEM to characterize fiber degradation under a prolonged exposure to an alkaline environment. The outcome captured surface irregularities (as shown in Figure 3), while in some regions, complete degradation of the fibers was evident.

Contrary to the studies that confirmed the degradation of PET fibers in alkaline environments, Ochi et al. [74] concluded that recycled PET fibers undergo negligible degradation after 120 h in an alkaline environment at 60 °C. This was quantified through direct tensile tests on individual fibers. This conclusion should be considered cautiously, as the alkaline exposure of the tested fibers may not have been long enough to relate the results to long-term durability considerations. Regardless, there is sufficient evidence in the literature to conclude that PET fibers can undergo some level of degradation in the concrete environment, which is a major limitation to the fiber’s reinforcing potential. PET fibers can have variable chemical and mechanical properties, depending on their manufacturing techniques. Similar to other polymeric fibers, the fiber-matrix bond of polyester fibers is reported to be only mechanical in nature [16].

### 2.8. Acrylic Fibers

**Figure 4:**
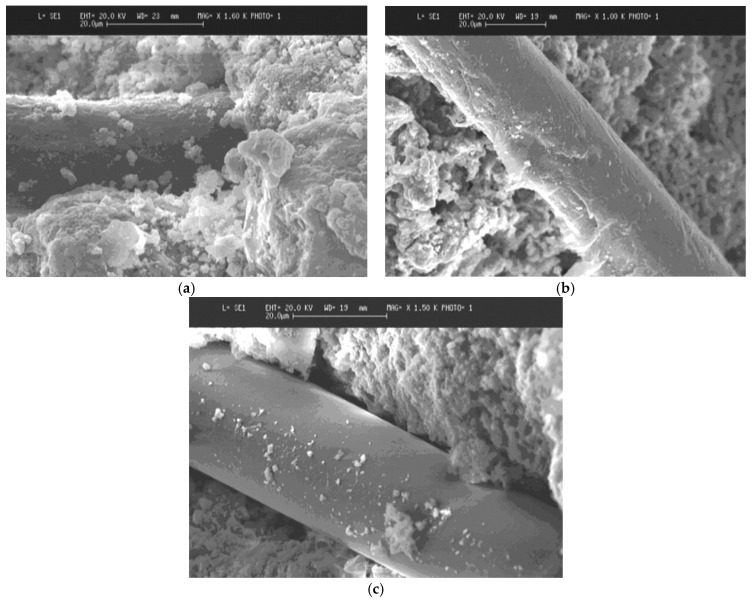
SEM images of (**a**) nylon, (**b**) acrylic, and (**c**) polypropylene fiber surface [76].

Early forms of acrylic fibers exhibited low strength and elastic modulus, as well as poor resistance to acids and alkalis, which limited their applications in concrete [18]. However, the new generation of acrylic fibers have shown little to no sensitivity to the alkalinity of concrete. Some research studies have reported small long-term sensitivity to alkaline environments, especially at higher temperatures [47], while others have reported that acrylic fibers are not sensitive to chemical degradation [75,76]. Hahne et al. [77] studied the performance of FRC made with high-strength PAN fibers. The study explored high-strength acrylic fibers of different lengths (i.e., 6–24 mm) and diameters (i.e., 18–104 micrometers), as well as strengths (up to 1000 MPa) and elastic moduli (up to 19.5 GPa) for their fiber-matrix bond properties. It was reported that acrylic fibers form a satisfactory bond with the concrete matrix, due to their irregular cross-sectional shapes. Confirming this assessment, Jamshidi and Karimi [76] exploited 3–4 mm fibers and found that acrylic fibers, similar to nylon fibers, form a stronger bond with the concrete matrix, in comparison to PP fibers, partly due to the formation of cement hydration products on the fiber surface, as illustrated in the SEM image provided in Figure 4 [76].

### 2.9. Aramid Fibers

A limitation of aramid fibers for use as a concrete fiber is the lack of clarity in the literature about the level of strength degradation of this type of fibers in the concrete environment [8]. Uomoto and Nishimura [78] found that the sensitivity of aramid fibers to chemical deterioration has a correlation to the method used for manufacturing the fibers. The cited study reported that aramid fibers that were acid spun (i.e., Kevlar) underwent degradation at high temperatures (80 °C and above) in acidic, alkaline, and distilled water solutions. Aramid fibers that were not acid spun (i.e., Technora) had much better chemical durability in similar solutions. Although the degradation of Technora aramid was an issue at high temperatures, such temperatures are not expected to be encountered in most concrete applications. Derombise et al. [79] studied the alkali resistance of Technora aramid fibers and reported that, despite small amounts of chain degradation after alkali exposures, the fibers retain nearly all of their mechanical properties. It is important to note that the tests were performed with pH values up to 11, while concrete provides an environment with higher pH values, which can exacerbate the alkali deterioration of the fibers. Uomoto and Nishimura [78] reported that aramid fibers were capable of retaining 90%, 60–85% and 45% of their strength after long-term aging in an alkaline, acidic, and ultraviolet exposure environment, respectively. Additionally, aramid fiber-reinforced polymer (AFRP) showed increased alkali resistance compared to monofilament aramid fibers [78]. Overall, the available studies suggest that aramid fibers can be sensitive to alkali degradation, however, if the fibers are not acid spun and high temperatures are not anticipated through the service life of FRC products, alkali degradation of the aramid fibers in concrete is not expected to be an issue. Kevlar fibers are reported to have a weak bond with the concrete matrix due to their smooth surface, inert nature, and high crystallinity [80]. To address this issue, Zhang et al. [81] conducted a chemical treatment on Kevlar fibers and observed that treated fibers can have a more roughened surface (and a better fiber-matrix bond) in comparison to untreated fibers.

### 2.10. Glass Fibers

**Figure 5:**
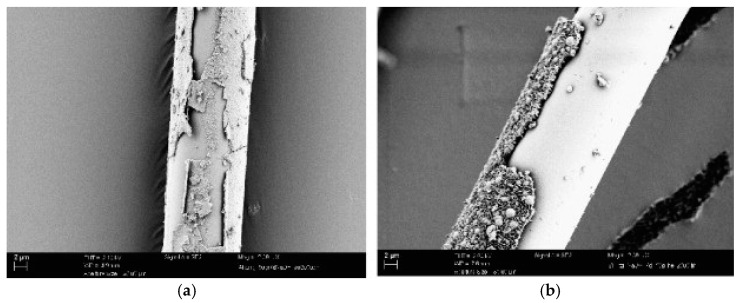
SEM images of glass fibers after alkali exposure: (**a**) silica glass fiber and (**b**) basalt glass fiber [84].

Despite superior mechanical properties, the main limitation of glass fibers in cementitious composites is their chemical sensitivity to alkaline environments. The alkali degradation of non-alkali resistant glass fibers are well documented in the literature [78,82,83]. Wu et al. [83] studied the durability of basalt and silica glass fibers after exposure to acid, alkali, and salt solutions. The study found that both basalt and silica glass fibers undergo full deterioration and retain none of their original strength characteristics after an extended exposure, noting the fact that the deterioration mechanism of basalt and silica glass fibers in concrete is similar, owing to their similar chemical compositions [83]. In the cited study, both fibers showed better resistance to salt solutions, although an approximately 40% loss in the tensile strength was recorded. The alkali deterioration was characterized by the pitted fiber surface. Similarly, Scheffler et al. [84] reported the formation of a shell around the fibers after 7 days of immersion in 5% NaOH solution, as shown in Figure 5. Such a deterioration was found to sacrifice the effective cross-section (and associated strength) of the fibers.

To reduce the degradation of glass fibers in alkali environments, zirconium oxides are added to the glass fiber production process to produce alkali-resistant (AR) glass fibers. The degradation prevention provided by the presence of zirconium oxides in glass fibers is because the Zr–O bonds are stable under alkali attacks, in contrast to the Si–O bonds, which break in the presence of hydroxides. This leads to a zirconium dioxide protective layer on the exposed fiber surface, serving as a barrier to prevent possible fiber breakdowns [18]. Adding zirconium oxides to glass fibers has become a common practice for the modification of silica glass fibers used in the concrete industry. Basalt glass fibers, however, have been subject to fewer research studies and are less common. Among the limited studies on AR basalt glass fibers, Lipatov et al. [85] can be highlighted. Despite the increased stability of AR glass fibers in alkaline environments, there is sufficient evidence that they undergo some level of strength degradation in concrete.

Based on the literature [86,87], AR glass FRC loses strength and ductility in tension and flexure as time progresses in natural weathering, underwater, and accelerated aging environments. The strength loss depends on pH, temperature, and chemical composition of the AR glass fibers and the concrete matrix, as well as the exposure condition [88,89]. ASTM C1666 [29] includes minimum specifications for AR glass fibers to be used in cementitious matrices. It can be noted that minimum strength retention values (expected after four days in hot water) are only 25% for water dispersible strands and 35% for integral strands when considering the lower bound of 1.0 GPa, as the initial fiber tensile strength. This lack of stringency in the standard shows that AR glass fiber strength degradation can be relatively large, while the fibers are still considered alkali-resistant. This will be discussed separately for silica and basalt glass fibers in the following sections.

#### 2.10.1. Silica Glass Fibers

It is generally accepted that AR silica glass fibers mixed in cementitious matrices lose some of their reinforcing effectiveness over time because of their chemical sensitivity to the alkaline environment, as explained in the previous section [84,90]. In order to help improve the long-term performance of AR glass FRC, Song et al. [91] investigated modifying the binder with a partial replacement of ordinary Portland cement with calcium sulfoaluminate cement. The study found that the proposed method greatly improves the long-term performance of the composites. After 10 years of aging, the modified composites retained substantial ductility compared to the control specimens, which showed no post-crack residual strength after 10 years of exposure. The cited study clearly reflects that, if proper mixture designs are used, glass fiber degradation can be mitigated. This can involve the use of pozzolans, such as silica fume, metakaolin, Class C and F fly ash, and pulverized borosilicate glass (also referred to as E glass).

In addition to adding zirconium to the chemical structure of glass, applying alkali-resistant sizing to the filament surface during production, or changing the chemistry of the concrete matrix, glass fiber strands can be impregnated with alkali-resistant and surface-bonding resins, such as epoxy and vinyl ester, to improve their long-term durability. These types of polymer-impregnated glass fibers are made into the macro concrete fibers that are relatively new to the concrete industry and are essentially miniature versions of GFRP rebars. The alkali degradation of GFRP macrofibers has not been fully described in the literature, however, due to the similarities that these fibers share with GFRP rebars, the research related to the durability of GFRP rebars can be cautiously extrapolated to evaluate the long-term durability of GFRP macrofibers.

The investigations that utilized accelerated aging techniques report concerns about the durability of glass-based fibers in concrete. Significant amounts of degradation and strength loss have been found, especially under high temperature and aggressive chemical environments [92,93,94]. The past studies sparked major concerns in the concrete industry about the level of safety provided by the structures that use GFRP, as primary reinforcement. These concerns motivated several case studies and critical reviews to characterize the level of GFRP strength degradation for in-service structures [95,96,97,98]. The listed efforts found that the degradation reported from accelerated aging tests on GFRP products largely overestimates the actual level of degradation in the field. Several case studies reported little to no GFRP degradation for in-service structures, owing to the effective protection provided by the polymer resin. The studies also concluded that the accelerated aging tests are not necessarily representative of the in-situ concrete condition, because of the use of elevated temperatures and the unlimited supply of hydroxyl ions [97].

Although several studies have focused on the bond properties of silica GFRP bars in concrete, limited studies are available concerning the bond properties of silica glass fibers in concrete, which can be highly different from the bond properties of silica GFRP bars, due to differences in their size and shape. In a study completed by Scheffler et al. [99], the pull-out properties of AR glass fibers with two types of sizing were investigated under quasi-static and high-rate (i.e., impact) loading protocols. Upon measuring the local interfacial shear strength and critical energy release rate, it was found that regardless of the sizing type, the interfacial friction stress undergoes a reduction when a high-rate load is applied, mainly because of smoothing the surface asperities of AR glass fibers. The study concluded that it is possible to control the pull-out behavior of AR glass fibers through adopting an appropriate sizing, which can be significantly helpful to adjust FRC’s post-peak mechanical properties.

#### 2.10.2. Basalt Glass Fibers

With regards to chemical durability, basalt glass fibers show an alkali degradation similar to that of E glass fibers [83,100]. In order to overcome this drawback, a range of methods have been examined in the literature, in addition to developing AR basalt glass fibers. Rybin et al. [101] studied the alkali resistance and mechanical properties of basalt glass fibers coated with zirconyl chloride octahydrate. The study found that the surface-coated fibers undergo delayed strength degradation under alkali exposure. This was also attributed to the surface coating thickness and density. Lipatov et al. [85] investigated the addition of zirconium oxides to basalt fibers during their manufacturing process. The study found that the solubility limit of zirconium in basalt glass is 7.1%, i.e., much less than that of silica glass. Despite the inability to reach high zirconium content during manufacturing, the AR basalt glass fibers with 5.7% zirconium content showed an alkali degradation (in terms of weight loss) similar to the AR silica glass fibers with 18.8% zirconium content. The strength degradation of the AR basalt glass fibers was substantially higher than that of the AR silica glass fibers, however, the compressive, tensile, and flexural strengths of the hardened mortars prepared with the basalt glass fibers that had an optimal zirconium content remained similar to those of the mortars prepared with the AR silica glass fibers [85].

Mingchao et al. [102] tested the chemical resistance of AR basalt glass fibers by boiling them in distilled water, salt solution, and acid solution. It was reported that the AR basalt glass fibers undergo stiffness and strength degradation in acid solution. In alkali solution, however, their stiffness was mostly maintained, but their strength underwent a gradual decline. In recent years, similar to AR silica glass fibers, filaments of basalt glass fibers have been impregnated with alkali-resistant polymer resins to create BFRP macrofibers. The same long-term durability aspects discussed in the silica glass fiber section of this review study are valid for basalt glass fibers as well. Considering the lack of studies focusing on basalt glass fibers, this extrapolation can be justified, especially due to the fact that similar alkali-resistant polymer resins are used to impregnate both GFRP and BFRP.

According to Jiang et al. [103], the SEM images of the concrete mixtures reinforced with both micro and macro basalt glass fibers reveal that chopped basalt glass fibers are densely covered with hydration products after seven days of curing, which creates a satisfactory bond with the concrete matrix. However, after 28 days of curing, the SEM images show a gap between the individual fibers and the concrete matrix, implying the possibility of debonding in later ages. In a separate effort, Arslan [104] reported the presence of a partial bond between the macro basalt glass fibers and their surrounding concrete, which contribute to increasing the mechanical strength of FRC. Furthermore, the cited study reported that all the fibers failed by pull-out and no fiber rupture was observed. This can be attributed to the high tensile strength of basalt glass fibers, outperforming the fiber-matrix bond strength.

## 3. Workability

Concrete is the most commonly used material in the construction industry. One of the principal reasons for such a widespread application is the concrete’s workability in the fresh state, making it possible to form several shapes in various sizes without needing any special treatments [105]. Therefore, properly selecting the FRC ingredients and adjusting their proportions to achieve the desired workability is critical. In particular, the shape, surface area, and dosage of the fibers, along with their water absorption capacity, are among the main deciding factors, which are explored with necessary details in this section.

### 3.1. Polypropylene Fibers

Mohod [106] reported that PP fibers tend to form undispersed clumps and significantly reduce the slump at volumes above 1.0%. However, this was found highly dependent on fiber dimensions and the mixture design. Dopko et al. [5] had a similar observation, indicating that PP macrofiber additions above 1.0% fiber volume greatly reduce the workability of FRC. In a comparison between PP and nylon fibers (used with a similar dosage in concrete), it was reported by Heo et al. [107] that the addition of PP fibers has a notable effect on the workability of FRC. The cited study also reported that using long PP fibers can further decrease the workability.

### 3.2. Nylon Fibers

Nylon fibers are hydrophilic and can absorb a small amount of water during the mixing process [16]. Several studies have found this feature beneficial to the dispersion of nylon fibers, comparing to PP fibers. However, at higher volume dosages, the water absorption capacity of the nylon fibers may adversely affect the mixture’s workability, due to the excessive absorption of mixing water. Yap et al. [49] noted that the workability of nylon FRC was less than that of PP FRC at the same fiber content in lightweight concrete. This could be due to the fact that, with a fiber volume of up to 0.75% tested in the cited study, the nylon fibers absorbed a significant amount of water, decreasing the workability of the mixture. Khan and Ali [50] reported that 50 mm-long nylon fibers dispersed at fiber volumes close to 1.5% reduce the slump to almost 30% of the slump obtained for the control mixtures that contained no fiber.

### 3.3. Polyvinyl Alcohol Fibers

When PVA fibers are used in concrete, the workability of concrete drops due to the PVA fiber’s water absorption. Hossain et al. [108] evaluated the effect of PVA addition on fresh and rheological properties of self-consolidating concrete (SCC). The cited study observed that PVA microfibers greatly reduce the flowability and passing ability of SCC. In particular, it was reported that the addition of PVA fibers decreases the plastic viscosity of SCC. Additionally, as the fiber content was increased further, the viscosity witnessed a higher reduction. Compared to micro and macro steel fibers, PVA fibers were found to have a more pronounced effect on reducing the flowability and passing ability of SCC. Shafiq et al. [109] reported the need for an increased water-to-cement ratio and a sufficient dosage of superplasticizer to meet the target slump for PVA macrofiber mixtures. The cited study was able to achieve satisfactory workability characteristics with 3.0% PVA fiber volume. Dopko et al. [5] reported difficulties when mixing macro PVA fibers in concrete at volumes over 1.0%, indicating that the fibers tend to re-aggregate and form clumps once a critical volume is reached. The cited study also found that PVA fibers decreased workability and caused dispersion issue at the same fiber volume of PP fibers, even though the PP fibers had a higher aspect ratio than the PVA fibers.

### 3.4. Polyolefin Fibers

Limitations in the fresh state as a result of adding PO fibers are similar to those previously discussed for PP fibers. PO fibers with surface indentations may further decrease the workability compared to smooth PO fibers, mainly because of the increased surface area per fiber. Several studies have shown that PO fibers can be used in SCC mixtures [110,111]. No significant detrimental effects to workability, however, have been reported in the literature for PO fibers when added in low volumes. Alberti et al. [110] indicated that macro PO fibers with the length of 50 mm can mix well in the SCC at volumes up to 1.0%. However, it should be noted that the cited study utilized a high water-to-cement ratio (i.e., 0.5) to improve workability. Zaroudi et al. [111] reported that the addition of PO fibers with more than 1.0% of volume fraction significantly reduces the slump flow of SCC. Smirnova et al. [112] compared two methods of adding PO fibers to the mixture and concluded that the addition of PO fibers to the fresh concrete and further mixing it for 5 min lead to insufficient dispersion and the agglomeration of fibers. The proposed solution was mixing fibers with the dry constituents (aggregates and cement) for one minute prior to the addition of water and superplasticizer. Noting that the macrofibers are often better dispersed in the concrete matrix compared to microfibers, the maximum volume fraction of macro PO fibers in concrete can be higher than that of micro PO fibers [112].

### 3.5. Carbon Fibers

Macro carbon fibers are uncommon since carbon fibers tend to break into shorter lengths during the mixing process because of their brittle nature [113]. In particular, the presence of coarse aggregates can increase the level of carbon fiber damage while mixing, however, such damage can be lessened by using appropriate mixing procedures and additives, such as methyl cellulose and superplasticizer, to further disperse the fibers with minimal mixing requirements [114]. The upper limit of carbon fiber dosage for conventional mixing has been found to be 1.0% by volume, due to the fiber’s high aspect ratio and specific surface [8], although higher volumes can be accommodated with modified mixing procedures and including admixtures [115]. Dopko et al. [116] reported adequate workability and dispersion of carbon microfibers in the FRC mixtures that contained up to 0.5% carbon fiber volume. This was achieved by the addition of superplasticizer and a modified mixing procedure to increase the mixing energy.

### 3.6. Polyethylene Fibers

Zhang and Li [117] reported that the addition of PE fibers decreases the workability of the FRC that contains fly ash and silica fume by reducing both slump and slump flow. PE macrofibers with lower strength and modulus of elasticity, similar to those of PP and PO, have been reported to mix sufficiently well into a normal FRC matrix at volumes up to 4.0%. This is a relatively high fiber volume and it should be noted that a high water-to-cement ratio was utilized in the cited study to help with mixing. High volumes, i.e., in the range of 2.0–4.0%, of high aspect ratio HSPE fibers were used by Yamaguchi et al. [118]. Such fiber volumes did not cause any slump issues because of utilizing a high superplasticizer dosage and a high shear force double-axis mixer.

### 3.7. Polyester Fibers

Although most of the past studies incorporated 1.0% PET fiber by volume (or lower) into FRC, PET macrofibers were reported to mix well in concrete at 1.5% or even up to 3.0% [74,119]. It must be noted that the cited studies utilized water-to-cement ratios equal to (or above) 0.55, leading to workable mixtures.

### 3.8. Acrylic Fibers

Addition of PAN fibers from a low volume up to 2.5% was reported to decrease the workability of FRC to the extent that the water-to-cement ratio has to be increased substantially. Superplasticizers can also be needed to accommodate the fiber addition, especially with using low-diameter fibers [77,120,121].

### 3.9. Aramid Fibers

Nanni [122] investigated different volumes of AFRP macrofibers dispersed in concrete. AFRP fibers include hundreds of aramid microfibers bound together by resin to form a single macrofiber. The cited study found that AFRP fibers significantly decrease the apparent workability and slump of the concrete. Thus, 2.5% was recommended as the maximum volume fraction of AFRP fibers that can be incorporated into FRC with conventional mixing procedures [122].

### 3.10. Glass Fibers

#### 3.10.1. Silica Glass Fibers

AR glass fibers can be used in the FRC made with conventional mixing procedures. However, it has been reported that high fiber volumes are difficult to achieve when using glass fiber filaments in concrete with conventional mixing procedures, because such fibers tend to disperse into the matrix unevenly, therefore, an increase in water-to-cement ratio or additional mixing becomes required [18]. The additional mixing can potentially damage the fibers and compromise their long-term performance [8]. It should be noted that the effect of AR glass fibers on the workability of conventionally-mixed concrete is highly dependent on the aspect ratio and surface area of the fibers, which can be drastically increased for filament strands compared to integral strands. The study by Ghugal and Deshmukh [123] reported that AR glass microfibers (up to 4.5% of cement weight) were mixed into the FRC that contained coarse aggregates with no mixing difficulties. The cited study employed a high water-to-cement ratio of 0.51 to increase workability, but there was no indication of using water-reducing admixtures.

#### 3.10.2. Basalt Glass Fibers

Ayub et al. [124] studied how the addition of high volumes (up to 3.0%) of basalt microfibers affects the workability of pozzolanic concrete (made with high-range water-reducing admixtures) and reported no mixing problem. Noting that this was a high microfiber content, achieving a satisfactory slump highlighted that with a proper mixture design and use of admixtures, as well as a high-energy mixer, high volumes of micro glass fibers can be incorporated into the FRC that contain coarse aggregates. In the case of basalt macrofibers, Arslan [104] reported no workability issues when using up to 3.0% of this type of fiber in concrete. This was further supported by reviewing the SEM images that showed basalt macrofibers disperse well in the concrete mixture, contrary to silica glass fibers that tend to form flocculation.

Since basalt glass fibers have a density relatively similar to that of the concrete matrix, BFRP macrofibers have been reported to mix well (at volumes up to 4.0%) in concrete using conventional mixing procedures, compared to most other fibers [125]. In a separate study completed by Branston et al. [126], BFRP fibers were found to clump at 2.0% volume, however, no superplasticizer had been used. For the SCC with a maximum aggregate size of 16 mm, it has been reported that BFRP macrofibers with an aspect ratio of 65 are detrimental to flowability at volumes over 1.15%, likely due to the stiffness and size of the fibers [127].

## 4. Pre-Peak Mechanical Properties

The engineering properties of the FRC used for structural design are often derived from pre-peak mechanical properties, where strength and modulus of elasticity are determined. It is generally accepted that the tensile and flexural strengths of a plain concrete is approximately 10% and 15% of the concrete’s compressive strength, respectively [105]. To improve both tensile and flexural strengths, fibers are added to the concrete mixtures in a wide variety of applications. When randomly dispersed fibers are incorporated into the concrete matrix, they act as reinforcement agents against tensile stresses. The main pre-peak mechanical properties of the FRC made with various fiber types are explored in this section.

### 4.1. Polypropylene Fibers

There are some inconsistencies in the literature as to whether or not PP fibers notably affect the strength parameters of FRC prior to crack propagation. Some studies have reported that the compressive strength of FRC increases or is not affected by the addition of macro PP fibers, but the tensile strength is significantly improved at volumes below 0.55% [128,129]. On the other hand, Cifuentes et al. [40] indicated that PP fibers increase both compressive and splitting tensile strengths of FRC. Some other studies found that low volumes of macro PP fibers had negligible impact on the flexural strength of concrete [130]. Ramezanianpour et al. [131] reported that the addition of PP fibers reduces the concrete’s compressive strength, but increases both splitting tensile and flexural strengths up to 40% and 10%, respectively. This trend was reported to be maintained until a fiber dosage of 0.7 kg/m^3^ is reached. The inconsistences among the available studies regarding the ability of PP fibers to increase pre-peak strength properties can be attributed to the variations in the fiber’s dosage, geometry, and mechanical properties, as well as the characteristics of the concrete matrix. Studies have found that recycled PP fibers can deliver similar mechanical properties, while averting fiber degradation in concrete [42,132].

### 4.2. Nylon Fibers

Song et al. [133] observed that the addition of nylon and PP fibers can increase the compressive strength and splitting tensile strength of FRC, where nylon fibers provided a better performance in comparison to PP fibers, owing to their higher tensile strength and elastic modulus. Yap et al. [49] also compared nylon and PP fibers, in terms of the compressive, splitting tensile, and flexural strength of FRC, and reported that, although multi-filament PP fibers had a higher flexural strength, the addition of nylon fibers improved the compressive and tensile strengths further. On the other hand, Zia and Ali [134] found that a 5.0% addition of nylon fibers (by weight of cement) decreases the compressive and splitting tensile strengths of FRC by more than 30% and 10%, respectively. Ozsar et al. [135] reported that nylon microfibers are more effective in increasing the splitting tensile strength of the mixtures with low water-to-cement ratios. This trend was noted to be reversed for nylon macrofibers.

### 4.3. Polyvinyl Alcohol Fibers

The studies on the use of PVA fibers have reported different results regarding their effects on the mechanical properties of FRC. In particular, it has been found that even low volumes of micro PVA fibers can reduce the compressive strength of FRC significantly [136]. On the other hand, Ahmad and Umar [137] reported that the PVA fiber addition of up to 0.3% of SCC volume contributes to increasing the compressive strength. Noushini et al. [138] showed that 0.25% PVA fiber addition increases the compressive and splitting tensile strengths of FRC, while any further increase in the fiber content can have counter effects on the strength. The splitting tensile and flexural strengths of FRC have been reported by several studies to remain the same or experience an increase with the addition of PVA fibers [5,108,109,137]. However, Yeganeh et al. [136] reported a drop in the flexural strength of FRC, although an increase in the splitting tensile strength was noted. In the absence of any explanation for this observation in the cited study, it is expected that the low water-to-cement ratio of 0.3 hindered the dispersion of PVA fibers in the mixture, especially given the water absorption characteristics of PVA fibers.

### 4.4. Polyolefin Fibers

Similar to other low-strength synthetic fibers, addition of low volumes of PO fibers to concrete does not have a significant effect on the mechanical properties of FRC. Alberti et al. [110] reported that, for low PO fiber volumes, only the tensile strength slightly increases, while for high PO fiber volumes, the compressive strength decreases slightly, and tensile strength increases substantially. Zaroudi et al. [111] observed that by increasing the fiber content to 1.25%, both compressive and splitting tensile strengths experienced improvements. They, however, both started decreasing upon adding to the fiber content, beyond 1.25%. Furthermore, the flexural strength of FRC was reported to increase with increasing the fiber content [139]. Alani and Beckett [140] investigated the performance of PO fibers (in comparison to hooked end steel fibers) for slab-on-ground reinforcement applications. It was found that the surface-embossed PO macrofibers can provide benefits similar to steel fibers. The volumetric dosage corresponding to the equivalent performance of PO fibers was about one-third higher than that of steel fibers. The study, however, showed that high-tenacity macro synthetic fibers have the potential to be used as the primary reinforcement in certain slab-on-ground applications. Similarly, Alberti et al. [110] described a case study, in which the conventional reinforcing bars of a concrete water pipeline casing had been completely replaced with 5 kg/m^3^ PO macrofibers. This led to a satisfactory outcome, as only small tensile stresses were anticipated in the concrete. By eliminating conventional bars, the construction cost and time of construction were both significantly reduced.

### 4.5. Carbon Fibers

Carbon fibers can improve the mechanical properties of cementitious composites if a sufficient volume is included. The extent of improvement is proportional to the strength and modulus of elasticity of the carbon fibers used. Stronger and stiffer carbon fibers more effectively increase the strength properties, while the weaker ones more likely contribute to enhancing the toughness. Among the limited studies available, Yao et al. [3] tested the FRC made with 0.5% volume high-strength micro carbon fibers and found that the fiber addition increased the compressive strength, splitting tensile strength, and modulus of rupture by 14%, 19%, and 9%, respectively. Chen et al. [141] reported that the addition of carbon fibers increases the compressive strength of the concrete and the best result is achieved when carbon fibers are used at 1.0% of weight of cement, although the cited study did not investigate the effect of higher fiber contents.

Dopko et al. [116] tested varying volumes of carbon microfiber, accelerating admixture, and shrinkage-reducing admixture for their effects on the compressive and splitting tensile strength of FRC. The study found that increasing the carbon microfiber volume generally increases the 24-h compressive and splitting tensile strengths of FRC. The presence of 0.3% carbon microfiber also increased the 7-day compressive and splitting tensile strengths by an average of 9.6% and 22.8%, respectively. On the other hand, Chen and Chung [142] reported that, although the addition of carbon fibers increased the flexural strength, the compressive strength of the specimens decreased, most likely because of the increased amount of air content, originating from fiber addition. It should be noted that the cited study used a relatively weak carbon fiber (with a tensile strength of 690 MPa), which can explain the reported findings.

### 4.6. Polyethylene Fibers

HSPE fibers have shown adequate reinforcing effects in concrete. In the limited studies available, mixtures with HSPE fibers (as low as 0.025%) have exhibited higher flexural strengths compared to those made with 0.1% fibrillated PP fibers [143]. In a separate effort, Yamaguchi et al. [118] explored 2% and 4% (by volume) of HSPE fibers for their effects on compressive, splitting tensile, and flexural strengths and reported an increase in all the strength values because of the HSPE fiber addition. The possibility of using recycled PE fibers for concrete reinforcement has also been investigated. Pešić et al. [66] studies the FRC that contained PE fibers made from recycled consumer products. The fibers used in the cited study had a relatively low yield strength (i.e., 12 MPa compared to 40–80 MPa, which is common for regular HSPE fibers) and a relatively low modulus of elasticity (i.e., 0.5 GPa compared to 0.9–1.1 GPa, which is common for regular HSPE fibers), mainly due to the recycling process. The study found that the FRC pre-peak strength properties were not significantly influenced by the addition of recycled fibers compared to the control mixture that contained no fibers.

### 4.7. Polyester Fibers

Research has shown that polyester fibers are capable of improving the mechanical properties of concrete. The bulk of research that has been conducted on PET fibers involves monofilament macrofibers made from recycled plastics; however, limited studies have also investigated non-recycled PET fibers. Swamy and Barr [144] tested 20 mm-long polyester fibers with a high aspect ratio at volumes up to 1.0%. The study found that the fiber addition increased the compressive, flexural, and splitting tensile strengths of the hardened composite by 5%, 7%, and 27%, respectively. Sivakumar and Santhanam [145] investigated polyester microfibers dispersed at 0.5% volume in a high-strength concrete matrix and determined that the compressive strength was not significantly affected by the addition of polyester fibers, but the elastic modulus, splitting tensile strength, and flexural strength were all improved. Recycled PET fibers have shown different effects on the concrete’s compressive strength, depending on their tensile strength, shape, length, and diameter. Kim et al. [70] compared the performance of recycled PET fibers (made through extruding shredded bottles) to non-recycled PP fibers. Both macro synthetic fibers were 50 mm long with similar aspect ratios, but the PET fibers were surface embossed, while the PP fibers were crimped. The cited study found that for both PP and PET fibers, compressive strength and elastic modulus slightly decrease with increasing the fiber volume. However, the ultimate flexural strength of FRC increased by adding to the fiber volume. Similarly, Borg et al. [119] investigated the FRC made with 0.5%, 1.0%, and 1.5% volume of recycled PET fibers that had been hand cut from waste bottles to two different lengths of 30 mm and 50 mm. Fibers of both lengths were either deformed or straight. The study found that the compressive strength is reduced when PET fibers are present in the mixtures, noting that the largest reductions occur for longer fibers mixed at higher volumes.

Fraternali et al. [71] studied the FRC mixtures that had been made with recycled PET macrofibers extruded from resins obtained from melting recycled bottle flakes. Three different recycled PET fibers were obtained each with different geometries and parent resins, giving them different mechanical properties. These three fibers were compared to non-recycled PP macrofibers with an embossed surface texture. The cited study reported that all the PET and PP fibers improved the compressive strength of FRC, although the straight PET macrofibers were able to provide a higher increase in the compressive strength than the embossed PP fibers. In a separate set of efforts, recycled PET fibers were found effective in increasing the flexural strength of FRC, noting that increasing their length can decrease their effectiveness [71,146]. When comparing the FRC products made with recycled PET fibers to those with PP fibers, a relatively similar increase in their mechanical properties is often recorded [70,146], although Rostami et al. [72] reported that PP fibers further enhance the flexural strength of FRC in comparison to PET fibers.

### 4.8. Acrylic Fibers

PAN fibers with different tensile strengths, lengths, and volumes are reported to have a negligible or negative effect on the compressive strength of FRC [77,120]. Mo et al. [120] used 12 mm-long acrylic fibers up to 0.2% and reported a 7–13% drop in the compressive strength. Furthermore, the cited study reported that the addition of PAN fibers increased both tensile and flexural strength of FRC, especially at a 0.1% dosage. This is in line with the results of Hahne et al. [77], which indicated that PAN fibers with a higher length can further improve the mechanical properties of PAN FRC.

### 4.9. Aramid Fibers

Zhang et al. [147] investigated aramid microfibers at volumes up to 1.5% in concrete. The study found that 0.5% fiber volume was able to slightly increase the compressive strength and elastic modulus of the composite, however, 1.0% and 1.5% fiber volume mixtures showed a reduced compressive strength and elastic modulus. Nanni [122] reported that AFRP fibers marginally increase the pre-crack flexural and splitting tensile strengths of FRC. The use of twisted macro Technora aramid fibers with 0.5 mm diameter and cut lengths of 30–40 mm was also investigated in the literature [148,149]. The studies found satisfactory results with twisted aramid macrofibers. Chan et al. [148] tested 30 mm and 40 mm long Technora aramid twisted macrofibers dispersed in concrete at 1.0% volume for their effects on the flexural response of steel reinforced concrete beams. The fibers did not significantly affect the compressive strength, but the peak flexural load in the beams was found to increase by about 9%. The fiber length did not cause significant improvements in the flexural test results. However, when compared to hooked end steel fibers, crack widths in the beams were smaller for aramid fibers up to the yield point of embedded steel bars.

### 4.10. Glass Fibers

#### 4.10.1. Silica Glass Fibers

Several studies have reported that the addition of glass fibers does not have a significant effect on the compressive strength of FRC, and in some cases, they were found to only marginally increase the compressive strength [104,123,150,151,152,153]. However, Khan and Ali [50] reported a drop in the compressive strength, which can be attributed to the relatively long glass fibers used in the cited study, although the silica glass fibers had a better performance than the nylon fibers tested. Söylev and Özturan [151] compared the effects of steel, glass, and PP fibers on the compressive strength of the specimens with two water-to-cement ratios of 0.45 and 0.60. The study, which employed both air curing and moist curing methods, found that the mixtures with glass and PP fibers had a rather similar performance, while steel fibers increased the compressive strength of the specimens.

In another study, Arslan [104] observed that macro basalt glass fibers have a more pronounced contribution to the compressive strength than silica glass fibers. Kizilkanat et al. [154] reported a similar observation for microfibers. Furthermore, Barluenga and Hernández-Olivares [150] indicated that a low-dosage addition of glass fibers (e.g., 600–900 gr/m^3^) does not change the flexural strength of FRC. However, by including more silica glass fibers, splitting tensile and flexural strengths of FRC improves [50,104,151,154,155]. Silica glass fibers have shown a higher contribution to splitting tensile and flexural strengths than nylon and PP fibers, while delivering marginally lower strengths in comparison to steel and basalt glass fibers [50,151]. Owing to the developments in the recycling industry, silica glass fibers can be extracted from GFRP, but they are in the form of fiber clusters that contain some remaining polymer and filler materials. Dehghan et al. [156] utilized this technology and examined the mechanical properties of recycled glass FRC and reported an increase in the FRC’s tensile strength, despite a decrease in its compressive strength.

#### 4.10.2. Basalt Glass Fibers

Different results have been reported on the pre-peak mechanical properties of basalt FRC. Yang and Lian [157] found that chopped water dispersible strand (micro) basalt fibers used at 0.3–0.5% volumes is an optimal dosage for increasing the compressive strength of FRC, while other studies reported that the addition of high volumes of micro and macro basalt fibers does not have a significant effect on the pre-peak mechanical properties of FRC [103,124]. On the other hand, Kabay [158] found that micro basalt fibers with 12 mm and 24 mm length dispersed at low volumes (2.0 and 4.0 kg/m^3^) decrease the compressive strength as the fiber volume increases for both normal and high-strength concrete. These contradictory results originate from different fiber characteristics and different concrete mixtures. While lower fiber contents can improve the packing of concrete, higher fiber contents, along with lower water-to-cement ratios, can signify the negative effects of fiber addition on the compressive strength of FRC. Studies have shown that basalt glass fibers enhance the splitting tensile and flexural strengths of FRC, regardless of the fiber content and fiber length [124,157,158]. Furthermore, Jiang et al. [103] found that longer fibers outperform shorter ones in improving splitting tensile and flexural strengths. Saradar et al. [159] investigated the flexural strength of 12 mm long basalt, steel, glass, PP, and PO fibers with 0.1% of volume fraction. The study reported that all the fibers increased the flexural strength of concrete, however, basalt and steel fibers made the highest contribution followed by PO, glass, and PP fibers. Similarly, other studies confirmed the superior splitting tensile and flexural performance of basalt glass fibers in comparison to silica glass fibers [104,154]. Branston et al. [126] investigated the effectiveness of chopped basalt filament microfiber bundles compared to BFRP macrofibers and concluded that filament basalt fibers can increase pre-crack flexural and compressive strengths in concrete, noting that the BFRP fibers decreased compressive strength and increased flexural strength at higher volumes. In a separate effort, Patnaik et al. [160] reported that BFRP macrofibers increased the flexural strength of concrete with increasing the fiber content.

## 5. Post-Peak Mechanical Properties

The expected performance and service life of reinforced concrete structures can be significantly affected with the occurrence of cracks, as investigated in several studies [161,162,163,164,165,166,167,168] at various length scales [169,170,171,172,173,174,175,176] with the consequences that can go beyond an individual structure [177,178,179]. The addition of fibers can address this issue, as the fibers act as load-transferring conduits over growing cracks in failed concrete regions, providing a residual (post-peak) strength, which in turn improves the concrete’s ductility and toughness. Such properties are critical, especially in response to the extreme events that create a high loading demand. Thus, this section has been devoted to investigating the post-peak mechanical properties of FRC based on various fiber materials and characteristics, such as diameter, length, and dosage.

### 5.1. Polypropylene Fibers

Several studies have reported significant increase in the post-crack residual strength and toughness of FRC, as a result of macro PP fiber addition [5,144,146,180]. Cengiz and Turanli [181] tested the toughness, energy absorption, and ductility of shotcrete panels reinforced with steel mesh, steel fibers, and high-performance PP fibers (with a low modulus). While the contribution of high-performance PP fibers to all the measured properties was found promising, increasing the PP fiber dosage beyond 1.1% decreased the ultimate load-bearing capacity, while negligibly increasing the energy absorption and flexural toughness characteristics. In general, a higher PP macrofiber content leads to a better post-crack performance, although, due to the low stiffness of PP fibers, residual strengths tend to be more positively influenced at larger deflections or wider crack openings. Based on the outcome of the past studies, it can be stated that, while there is some evidence that pre-crack mechanical properties of FRC can be modestly improved by PP fibers, the main advantages of adding macro PP fibers are realized after cracks are formed.

### 5.2. Nylon Fibers

Nylon fibers are utilized to enhance the post-peak characteristics of FRC. When dispersed in low volumes, nylon microfibers have minimal effects on the pre-crack mechanical properties. However, a higher toughness and a more ductile failure mode can be achieved with the addition of nylon fibers [91,182,183,184]. Zia and Ali [134] investigated the effects of addition of jute, nylon, and PP fibers on controlling the cracks. It was determined that, while the fiber additions improved the overall mechanical properties of FRC, PP fibers outperformed nylon fibers. This superior performance of PP fibers in comparison to nylon fibers was also observed in the total absorbed flexural energy, where PP fibers caused a 100% increase, while nylon fibers led to a 68% increase. This trend is much more significant in the total absorbed energy during splitting tensile tests, which showed a 21% decrease for nylon FRC, whereas PP FRC recorded an 11% increase. Furthermore, it can be understood from the cited study that PP FRC has superior post-peak properties compared to nylon and jute FRC. In a separate effort, Ozsar et al. [135] investigated the use of both macro and micro monofilament nylon fibers in the concrete matrices with two different strengths. Comparing micro and macro nylon fibers, the study found that micro nylon fibers increased the compressive strength of the composite and were most effective in reducing plastic shrinkage cracks, while the macro nylon fibers increased the fracture energy and improved the post-crack performance of the tested mixtures.

### 5.3. Polyvinyl Alcohol Fibers

PVA fibers have tensile strengths in the same range as steel fibers, however, the elastic modulus of PVA fibers is less than 25% of steel, as reflected in Table 1. Thus, PVA fibers have the ability to only modestly increase the tensile and flexural strengths of hardened concrete, but they more effectively increase the toughness and ductility properties [109]. Many studies have reported that the addition of micro and macro PVA fibers increases the flexural toughness, flexural residual strength, energy absorption, ductility, and impact resistance of concrete. Furthermore, it has been stated that increasing the fiber volume has often made a positive contribution to the aforementioned properties [54,108,109,136]. Shafiq et al. [109] compared the pre-peak and post-peak mechanical properties of FRC containing 1.0–3.0% of PVA and basalt glass fibers. Additionally, the study replaced 10% of cement with metakaolin and silica fume and completed the same tests. It was found that, although basalt glass FRC delivers a marginally higher flexural strength than PVA FRC, the latter has a superior post-peak flexural strength to the extent that FRC with a 3% PVA fiber addition provides deflection-hardening properties. In a separate effort, Hossain et al. [185] investigated the performance of PVA and metallic micro and macrofibers in SCC. It was reported that the incorporation of both fiber types can greatly enhance the fracture energy of SCC mixtures. This enhancement exceeded a 300% increase in the fracture energy of the SCC made with PVA fibers, which was attributed to the molecular bond formed between the individual PVA fibers and the SCC matrix.

### 5.4. Polyolefin Fibers

Macro PO fibers are typically used to increase the post-crack residual strength of the concrete. They can improve post-crack ductility and limit crack growth, but due to their low modulus, they are often not as effective for low deflections or small crack widths as other fibers with higher elastic moduli [110]. Ramakrishnan [186] described the use of macro PO fibers in bridge decks and barrier rails. It was reported that the addition of fibers at 1.5% volume not only improves the impact resistance and toughness of the concrete, but provides a synergistic effect with the rebar, shifting the cracking pattern from a lower number of wider cracks to a larger number of narrower cracks, which effectively limits the ingress of corrosive agents into concrete. Alberti et al. [110] compared the post-peak properties of the FRC made with 3.0, 4.5, 6.0, and 10.0 kg/m^3^ PO macrofiber contents with the FRC made with 26 kg/m^3^ of steel fibers. The study stated that, regardless of fiber volume, toughness and ductility increase with the addition of PO fibers, providing improved residual strengths. From the fracture energy results, it was noted that the addition of PO fibers can increase the fracture energy of concrete up to 75% of steel FRC after a 1 mm deflection, i.e., 1/300 of the span length. However, when the deflection increases to 5 mm, i.e., 1/60 of the span length, PO fibers outperform steel fibers by 40%, proving the higher efficiency of PO fibers in high deflections. It should be noted that an admixture for improving the fiber-matrix bond had been used in the cited study for high-volume fiber mixtures.

### 5.5. Carbon Fibers

FRC made with 0.5% carbon, PP, and steel fibers were tested by Yao et al. [3] for their post-peak properties. Steel fibers drastically outperformed carbon and PP fibers in the residual flexural strength and flexural toughness. Carbon fibers were found to increase the concrete’s residual strength, especially at smaller deflections. However, FRC with PP fibers showed a higher residual flexural strength at larger deflections. In addition, Chen and Chung [142] found that the flexural toughness of the FRC made with 0.19% carbon fibers can witness an increase by more than 150%.

### 5.6. Polyethylene Fibers

Low-strength PE fibers are effective in increasing the post-crack flexural ductility, especially at large deflections [187]. Yamaguchi et al. [118] showed that the addition of high volumes of micro HSPE fibers to concrete significantly increases its toughness and extreme load resistance. Moreover, Soroushian et al. [143] reported that HSPE fibers provide an impact resistance comparable to fibrillated PP fibers at low volumes. In a separate study, Pešić et al. [66] investigated the pre-peak and post-peak mechanical properties of FRC with 0.40%, 0.75%, and 1.25% volume fraction of recycled high-density PE fibers. For this purpose, two series of fibers (with 23 mm and 30 mm length and 0.25 mm and 0.40 mm diameter, respectively) were used. The cited study reported that a satisfactory flexural toughness and residual strength can be achieved by adding the recycled HDPE fibers. The residual flexural strength was found to be higher for the FRC samples made with shorter fibers (i.e., from 25% to 45% of the flexural peak value) and lower for the FRC samples with longer fibers (i.e., from 13% to 32% of the flexural peak value). Furthermore, it was stated that recycled HDPE fibers can be effectively used in the structural concrete, as they exhibit post-peak mechanical properties similar to PP fibers.

### 5.7. Polyester Fibers

There are only a few studies on the post-peak properties of non-recycled polyester fibers, while there are several studies conducted on recycled PET fibers. Swamy and Barr [144] used 20 mm long non-recycled polyester fibers with a high aspect ratio at volumes up to 1.0% and reported a 100% increase in the impact strength of FRC compared to that of plain concrete. The incorporation of recycled PET fibers in concrete was also found to enhance the post-peak properties of concrete. In particular, it has been reported that PET FRC benefits from high toughness and ductility [70,119,146]. Kim et al. [70] investigated the ductility and ultimate flexural strength of the FRC containing 0.5%, 0.75%, and 1.0% recycled PET and crimped PP fibers (with the length of 50 mm). The cited study showed that recycled PET and PP fibers have a similar performance characteristic and the addition of fibers increased their ductility and ultimate flexural strength significantly. According to Fraternali et al. [146], PET fibers with a higher tensile strength can deliver a higher flexural ductility. Kim et al. [70] reported that the ductility of a full-scale beam made with PET FRC can increase up to 10 times of that of the reference beam with no fibers. Toughness of the PET FRC increased with increasing the fiber volume [119]. Additionally, longer fibers were determined to further improve the toughness characteristics compared to shorter fibers, mainly because of the increased fiber-matrix bond strength [119].

### 5.8. Acrylic Fibers

Ductility and post-crack residual strength are known to increase with the incorporation of PAN fibers into the concrete matrix [76,77]. Among the limited studies available, Fan [121] investigated the contribution of PAN microfibers to the post-peak mechanical properties of FRC. In particular, fiber volumes between 0.5% and 2.0% were tested for their influence on impact toughness. The study concluded that the addition of PAN fibers enhances the impact toughness of concrete up to 250%, noting that 1.0% volume of PAN fibers provided the highest improvement.

### 5.9. Aramid Fibers

Nanni [122] found that a significant increase in the concrete’s post-crack residual strength and toughness can be achieved by adding AFRP fibers. The study reported that AFRP fibers greatly outperform PP fibers, while providing benefits similar to steel fibers. It is important to mention that steel fibers are prone to losing a portion of their capacity with the progress of corrosion, while aramid fibers would not undergo any conventional corrosion. Abeysinghe et al. [188] tested twisted Technora fibers with a 40 mm length to investigate their contributions to the extreme load resistance of concrete panels. A 1.0% volume of aramid fibers was found to reduce crack widths and eliminate spalling from such exposures. In addition, a 15% increase in the toughness of RC beams reinforced with 1% of twisted Technora fibers was reported.

### 5.10. Glass Fibers

#### 5.10.1. Silica Glass Fibers

Regardless of fiber length and volume fraction, silica glass fibers are found to increase the concrete’s ductility and toughness [151,154,155]. Furthermore, splitting tensile and flexural energy absorption of concrete is reported to increase, despite the drop in compressive energy absorption [50,104]. Silica glass fibers have delivered a superior performance in increasing the toughness of concrete compared to nylon fibers, while their performance was similar to PP fibers. On the other hand, steel fibers significantly outperformed glass fibers [50,151]. Arslan [104] used a range of 0.5 kg/m^3^ to 3.0 kg/m^3^ of silica glass fibers to measure the fracture energy of a set of beam samples. It was reported that adding 1.0 kg/m^3^ silica glass fibers has the maximum efficiency by increasing the fracture energy up to 35%, compared to the plain concrete. It was also mentioned that the specimens with silica glass fibers achieved a higher ductility and energy dissipation capacity, compared to the basalt FRC specimens.

#### 5.10.2. Basalt Glass Fibers

Basalt glass fibers are known to be effective in enhancing the concrete’s post-peak mechanical properties. Jalasutram et al. [189] reported that the addition of basalt glass fibers to the concrete can increasingly augment the deformability and flexural toughness of basalt FRC. Furthermore, the addition of basalt fibers has been determined to be more effective in increasing the ductility and crack resistance of FRC, compared to silica glass fibers. This trend is reversed in fracture energy, where silica glass fibers outperform basalt glass fibers [104,154]. Branston et al. [126] compared the effectiveness of chopped basalt filament microfiber bundles to that of BFRP macrofibers (minibars) and concluded that the BFRP macrofibers had a much better post-peak performance. With testing a mixture made with 2.0% volume of 43 mm-long BFRP macrofibers under flexure, an outstanding post-crack performance characterized by high ultimate strength, high residual strength, and initial post-crack strain hardening, followed by gradual strain softening at high deflections, was recorded.

In a study performed on BFRP macrofibers [190], it was found that the ratio of the average post-crack residual strength to the first crack strength can reach 0.75 with 2.0% fiber volume and as high as 1.00 with a fiber volume of 4.0%. This clearly reflected that high volumes of BFRP macrofibers can provide superior post-crack performance in FRC products. Patnaik et al. [125] indicated that BFRP macrofibers control the crack widths better than high-tenacity macro PP fibers in the beams subjected to accelerated corrosion and then tested in flexure. This was attributed to the fiber’s increased stiffness and the superior bond properties between the impregnating resin and the concrete matrix. Patnaik et al. [160] reported that increasing the dosage of BFRP macrofibers in concrete further increases the post-crack residual strength of FRC. Furthermore, Patnaik et al. [191] investigated the addition of low volumes of BFRP macrofibers and high-tenacity PP macrofibers to the concrete used in bridge decks. The cited study found that BFRP macrofibers are more effective than high-tenacity PP macrofibers in controlling the crack width and increasing the ductility.

## 6. Shrinkage

Water plays a critical role to initiate and help with the hydration reactions required to achieve the desired fresh and hardened properties of concrete. However, the consumption or the loss of water can result in shrinkage, incurring tensile stresses that can exceed the relatively low tensile strength of the concrete matrix [192]. The formation of cracks expedites the transport of corrosive agents, mainly chloride ions and CO_2_, into concrete, endangering the durability of reinforced concrete structures [193,194,195,196,197]. Several methods have been suggested to help concrete withstand shrinkage-induced cracks, including the use of shrinkage-compensating cement [198,199,200] and/or fibers. When fibers are incorporated into a concrete mixture, even in low dosages, they can withstand the induced tensile stresses, preventing the formation of cracks that can endanger the life-cycle performance and durability of concrete structures. Thus, it is imperative to investigate and understand how various fiber characteristics contribute to lowering the shrinkage potential and minimizing the extent of cracks in the concrete matrix, especially at early age.

### 6.1. Polypropylene Fibers

PP fibers can highly reduce the drying shrinkage cracking of concrete by increasing the capacity of FRC to resist shrinkage-induced strains [201]. The available studies have shown that plastic shrinkage in concrete can also be limited by using PP fibers [202,203]. Furthermore, it has been reported that increasing the fiber dosage can help with reducing (or even eliminating) the shrinkage-induced effects by minimizing the number of cracks and their widths. Fibrillated PP microfibers have a relatively small diameter and high aspect ratio, making them more effective for controlling plastic shrinkage cracks in fresh concrete compared to PP monofilaments [202,204].

### 6.2. Nylon Fibers

Nylon fibers have found to be effective when it comes to restricting the propagation of drying and plastic shrinkage cracks in concrete. Nam et al. [205] substituted the natural fine aggregates with recycled aggregates and witnessed an increase in drying shrinkage. To resolve the issue, low volumes of nylon fibers (in the range of 0.1% to 0.5%) were added. The addition of nylon fibers was found to increase the resistance of the recycled aggregate concrete to shrinkage, even above the resistance of the natural aggregate concrete, which had no fibers.

### 6.3. Polyvinyl Alcohol Fibers

Both macro and micro PVA fibers are reported to be effective in controlling drying shrinkage cracks in concrete. It has been found that PVA fibers added to the concrete at relatively low volumes (below 0.5%) decrease the shrinkage-induced crack widths by 90% for microfibers and 70% for macrofibers. The PVA fibers did not affect the restrained drying shrinkage stress development rate and time of first crack generation. However, they controlled the crack widths once cracks initiated. In a separate investigation [206], pre-crack strength was found not greatly influenced by the addition of PVA fibers, but residual strength was positively impacted. Wongtanakitcharoen and Naaman [51] studied the unrestrained early-age shrinkage of the FRC made with 0.1% to 0.4% addition of PVA fibers. The cited study concluded that PVA fibers controlled the unrestrained early-age shrinkage by 34% (on average), providing an improved performance, compared to carbon and PP fibers with the same volume fractions.

### 6.4. Polyolefin Fibers

PO fibers of different lengths and aspect ratios are reported to be effective in controlling plastic shrinkage and thermal cracking in concrete overlays. Shorter fibers proved to be most effective for such applications at the same volume dosage [207]. Yousefieh et al. [208] found that controlling the drying shrinkage in the FRC made with a 1.0% PO fiber content is not as effective as that with steel fibers, mainly due to the steel’s higher modulus of elasticity. However, the PO fibers were determined to have a better performance than the PP fibers. Furthermore, the crack initiation time was found to be delayed as a result of fiber addition.

### 6.5. Carbon Fibers

Limited studies have measured the effect of carbon fibers on the shrinkage of concrete. Carbon fibers have shown to be effective in reducing the restrained shrinkage and drying shrinkage cracking potential of carbon FRC [142]. Dopko et al. [116] tested the restrained drying shrinkage of FRC with 0.1%, 0.3%, and 0.5% carbon microfibers. The cited study concluded that, although carbon microfibers show negligible effects on the stress rate and magnitude caused by restrained shrinkage, they can efficiently control the crack opening potential by increasing the tensile strength of FRC. It was also stated that accelerating admixtures (ACC) have an adverse effect on the restrained drying shrinkage of carbon FRC, as captured by the strains recorded during the ring tests. On the other hand, shrinkage-reducing admixtures (SRA) were reported to show a great potential for controlling drying shrinkage-induced strains. It was also noted in the cited study that SRA can compensate for the negative effects of ACC on the drying shrinkage.

### 6.6. Polyethylene Fibers

Pešić et al. [66] investigated the FRC made with recycled PE fibers and found that the total number and the width of plastic shrinkage-induced cracks significantly decreased by the presence of fibers even at low volumes. The study reported that the crack reduction ratio ranges from 34% to 84% for the samples containing 0.40% to 1.25% recycled PE fibers, respectively. The unrestrained drying shrinkage of concrete was also investigated in the cited study. With a 10–15% drop in the strains recorded, it was stated that the reduction achieved was in the same range as that from PP and other synthetic fibers. In another study, Auchey and Dutta [209] investigated recycled high-density PE fibers. The study concluded that the FRC containing this type of fiber performed equal or better than that made with PP fibers in freeze-thaw conditions, suggesting that recycled high-density PE fibers can be a secondary reinforcement alternative for resisting shrinkage and temperature gradient effects.

### 6.7. Polyester Fibers

Recycled PET fibers are reported to be effective in controlling shrinkage-induced cracks. According to Borg et al. [119], recycled PET fibers can reduce plastic shrinkage cracking under accelerated drying conditions, as well as reduce and delay crack opening under restrained drying shrinkage. Kim et al. [70] added that the time to crack formation under restrained drying shrinkage was elongated with increasing the fiber volume. Pelisser et al. [210] indicated that, among short PP, recycled PET, glass, and nylon fibers, the short PP fibers were best in controlling the crack initiation caused by plastic shrinkage, where the recycled PET and glass fibers showed a similar performance, while the nylon fibers had the weakest performance. This led to recommending short, recycled PET fibers as a promising substitute for PP fibers to limit plastic shrinkage.

### 6.8. Acrylic Fibers

The addition of PAN fibers minimizes the drying shrinkage cracking potential, regardless of the volume fraction of the fiber, noting that including higher volumes leads to a better performance [77,120]. Fan [121] investigated the effects of PAN fibers on the autogenous shrinkage of FRC. It was reported that a 21.7%, 39.1%, 26.1%, and 17.4% reduction of autogenous shrinkage-induced strains can be achieved in the FRC made with 0.1%, 0.5%, 1.5%, and 2.0% PAN microfibers, respectively. This improvement can be attributed to the contribution of PAN fibers to modifying the FRC’s pore structure, which was verified through mercury intrusion porosimetry analyses.

### 6.9. Aramid Fibers

Zhao et al. [149] investigated 30 mm long Technora aramid macrofibers for their contribution to limiting plastic shrinkage cracking and restrained drying shrinkage at volumes between 0.2% and 1.2%. The addition of 0.4% volume of aramid fibers (and above) was found to eliminate plastic shrinkage cracks. Furthermore, the cited study reported that the addition of 0.8% volume of aramid fibers (and above) can decrease drying shrinkage strains by 15%.

### 6.10. Glass Fibers

#### 6.10.1. Silica Glass Fibers

Small additions of glass fibers have been found effective in controlling shrinkage-induced cracks. Barluenga and Hernández-Olivares [150] studied drying shrinkage under both free and restrained conditions. The cited study found that even the addition of very small amounts of micro glass fibers (600 gr/m^3^) has a significant contribution to reducing the cracked area and the length of cracks in both regular concrete and SCC. It was also concluded that, although increasing the fiber content increases the concrete’s ability to withstand drying shrinkage, the efficiency of fibers begins to diminish beyond a certain dosage [150]. Soranakom et al. [211] indicated that the silica glass fiber addition enhances the concrete’s crack resistance against drying shrinkage by delaying the time of cracking and lowering the crack width. In the case of restrained plastic shrinkage, Malathy et al. [212] tested different volume fractions of micro silica glass fibers and found that the fibers are very effective in controlling plastic shrinkage, even in the concretes containing silica fume. In a separate effort, Dehghan et al. [156] investigated the effect of recycled silica glass fibers on the drying shrinkage of FRC and concluded that the ability of concrete to withstand drying shrinkage is not improved with this type of fiber. Such an assessment was justified based on the low stiffness of recycled silica glass fibers.

#### 6.10.2. Basalt Glass Fibers

Branston et al. [126] tested the effects of filament dispersion and bundle dispersion of basalt fibers (up to 0.3% of concrete volume fraction) on controlling free and restrained plastic shrinkage. The cited study reported that basalt fibers are highly effective in improving the concrete’s ability to withstand plastic shrinkage by decreasing shrinkage-induced strains and limiting the crack growth. It was found that, regardless of the type, the addition of 0.1% volume fraction of basalt glass fibers can eliminate plastic shrinkage-induced cracks. This volume fraction was reported that can be further reduced by utilizing lower diameter filaments. In particular, filament dispersion was determined to deliver the best performance compared to bundle dispersion and BFRP minibars [126]. Consistent with the reported results, Saradar et al. [159] evaluated the early-age restrained shrinkage of various FRC mixtures and reported that the concretes containing PP and steel fibers had the lowest crack width in comparison to those made with basalt and silica glass fibers. As for the initiation of the first crack, it was found that the mixtures with basalt glass fibers had the earliest crack initiation time. Additionally, it was reported that, as the stiffness of the fibers increases, their flexural strength increases, while their ability to limit restrained shrinkage declines.

## 7. Extreme Temperature Resistance

In a hardened concrete, water can be found in two main phases: free water and bound water, while the latter is further categorized to physically bound and chemically bound water. When concrete is exposed to extreme temperatures, the water inside the concrete evaporates and if the entrapped vapor does not find a way out, it generates an internal tensile stress, which can eventually make the concrete implode and spall. One of the methods used to increase the concrete’s capacity to withstand extreme temperatures for an extended period of time is the incorporation of fibers. As discussed in Section 4, the addition of fibers enhances the tensile strength of concrete, which is desirable in resisting extreme temperatures. In addition, synthetic fibers have a relatively low melting point, which can provide an escape route for the entrapped vapor when needed. Noting all these aspects, it is important to understand the potential of each fiber type in enhancing the concrete’s performance under extreme temperatures.

### 7.1. Polypropylene Fibers

PP fibers have a relatively low melting point (from 160 to 170 °C [213]) in comparison to most other concrete fibers and should not be used in high temperature applications, such as autoclave curing [214]. The low melting point of PP fibers gives rise to their applications for spalling prevention during fires in concrete structures. As the fibers reach their melting point during a fire, they provide escape routes for the highly compressed gas caused from the vaporization of moisture inside the concrete [182]. As the water-to-cement ratio decreases, the need for PP fibers to prevent spalling increases. However, shorter fibers have shown a better contribution to fire resistance compared to longer ones.

### 7.2. Nylon Fibers

Nylon fibers have a melting point between 215 and 265 °C [215]. When exposed to extreme temperatures, ordinary concrete undergoes severe damage; however, experiments have shown that when nylon fibers are incorporated into a concrete mixture, they can reduce the damage and prevent spalling. Additionally, increasing the fiber content leads to an improved protection for FRC against fire and extremely high temperatures [107,182].

### 7.3. Polyvinyl Alcohol Fibers

The melting point of PVA fiber is reported to be between 220 and 230 °C [216,217,218]. Heo et al. [107] utilized PP, PVA, and nylon fibers with different lengths and volume fractions in concrete to evaluate their efficiency when exposed to elevated temperatures. The cited study found that PVA fibers were helpful in controlling the spalling and retaining the compressive strength of FRC. PVA fibers provided a better spalling control than PP fibers, however, nylon fibers outperformed PVA fibers, due to the presence of higher number of fibers per unit volume of nylon fibers. Moreover, it was reported that, for the concrete specimens that did not experience a severe damage by extreme temperature, PVA-containing samples retained a higher residual compressive strength than those with PP fibers [107].

### 7.4. Polyolefin Fibers

PO fibers have a (relatively) low melting point, which is reported to be 150 °C [219]. Hence, when exposed to fire, the PO fibers tend to melt quickly and make escape channels for water vapor. Therefore, they have the potential to enhance the concrete’s ability to prevent or delay spalling, while maintaining a higher strength after an extreme heat exposure.

### 7.5. Carbon Fibers

There are limited studies concerning the fire resistance of carbon FRC. Chen and Liu [220] compared the performance of carbon, steel, and PP fibers in high-strength concretes exposed to high temperatures (up to 800 °C). The cited study reported that the samples with no fibers experienced explosive spalling. The addition of carbon and steel fibers delayed the spalling, while the addition of PP fibers completely eliminated it. These observations were attributed to the behavior of fibers exposed to elevated temperatures. Carbon and steel fibers can delay initiation and propagation of microcracks and microdefects, owing to their (relatively) high elastic modulus. However, PP fibers melt due to their low melting point, generating evacuation channels for water vapor inside the concrete. Additionally, the cited study measured the residual compressive and splitting tensile strengths of the specimens exposed to extreme temperatures and found that concretes with carbon and steel fibers retained higher values of compressive and splitting tensile strengths, compared to those with PP fibers [220].

### 7.6. Polyethylene Fibers

PE fibers have a low melting temperature, i.e., 130 °C for regular HDPE fibers and 150 °C for gel spinned HDPE fibers [22,221]. Therefore, when PE FRC is exposed to extreme temperatures, the fibers tend to melt and generate empty channels, which can help with water evaporation, thus, improve the performance of concrete after fire exposure. Sukontasukkul et al. [222] states that the pre-peak responses of plain and FRC concrete were similar after fire exposure, but as for post-peak properties, exposure temperature and fiber type are the two main parameters that influence the performance of FRC, especially if the temperature goes above 400 °C [222].

### 7.7. Polyester Fibers

PET fibers have a low melting point of 160 °C, as reported by Sadrmomtazi and Tahmouresi [223]. Therefore, they tend to melt when exposed to high temperatures [74]. Increasing the PET fiber content enhances the concrete’s ability to withstand the negative effects of severe temperatures. Song et al. [224] found that lowering the fiber’s diameter can enhance the FRC’s potential to tolerate fire effects. In a separate study, Choi et al. [225] investigated the effect of PET fibers on reducing the spalling of high-strength concretes exposed to two different fire curves (i.e., RABT and ISO834) and reported that 0.2% addition of PET fibers can completely eliminate the spalling of concrete in both heat exposure conditions.

### 7.8. Acrylic Fibers

Mo et al. [120] investigated low volumes (below 0.2%) of acrylic microfibers in the lightweight oil palm shell concrete that contained ground granulated blast furnace slag. The study observed that the acrylic fibers were effective in preserving the concrete’s strength after heat exposure, owing to the low melting point of the fibers, allowing the entrapped gas to escape the concrete. The melting point of the PAN fiber was reported to be 145 °C in Mo et al. [120], while Moody and Needles [226] indicated that the PAN fiber’s softening point is between 190 and 250 °C.

### 7.9. Aramid Fibers

Aramid fibers are reported to start their tensile strength loss at 200 °C and they completely lose their strength when the temperature reaches 400–500 °C, which is a relatively high tolerance compared to other fibers [22]. Therefore, considering the concrete’s thermal conductivity, aramid fibers do not melt when exposed to fire, unless a long exposure to heat occurs. Consequently, aramid fibers can help FRC further maintain its strength through bridging the cracks even under fire.

### 7.10. Glass Fibers

#### 7.10.1. Silica Glass Fibers

Mirza and Soroushian [155] compared the performance of the FRC made with silica glass fibers to the plain concrete after exposure to high temperatures. It was reported that since silica glass fibers do not melt, they help the concrete maintain a (relatively) high flexural strength after heat exposure since the fibers prevent cracking initiated by the stresses from water vapor and subsequent cooling shrinkage.

#### 7.10.2. Basalt Glass Fibers

Basalt glass fibers have an outstanding thermal stability, which is reflected in a study conducted by Sim and Park [227]. In the cited study, the FRC mixtures that contained silica glass, carbon, and basalt glass fibers were heated for two hours at various temperature levels, including 100, 200, 400, and 600 °C. It was found that the mixtures made with silica glass and carbon fibers lose more than 40% of their strengths, while basalt FRC preserved 90% of its strength. Additionally, when the fibers were heated to 1200 °C, it was observed that silica glass and carbon fibers totally melted, while basalt glass fibers retained their geometry and mechanical integrity [227].

## 8. Synthesis and Recommendations

The addition of fibers can greatly alter many fresh and hardened properties of concrete, depending on the fiber’s chemical and physical characteristics. Based on several investigations reviewed in the current study, this section provides a synthesis of the most common trends and observations reported on the performance of fibers in each of the categories of stability and bond, workability, pre-peak and post-peak mechanical properties, shrinkage, and extreme temperature resistance. Additionally, to help the researchers and engineers in the concrete industry with the selection of fibers, the performance of each of the fiber types in each of the performance categories has been qualitatively ranked as weak (W), fair (F), good (G), or excellent (E) in Table 3. For the combinations that had very limited information available in the literature, not enough information (NI) has been listed.

### 8.1. Stability and Bond

Portland cement concrete provides a highly alkaline environment, with a pH value as high as 13.5, which protects steel rebars from corrosion. Such an environment is proven to cause deterioration in some fiber types. Therefore, ensuring their long-term stability is of paramount importance. Among the fibers investigated, both silica and basalt glass fibers have shown to be significantly degraded in the concrete matrix, reflecting the need to employ alternative methods to enhance the alkalinity tolerance of the glass-based fibers. Such methods range from the coating of the individual fibers with alkali-resistant materials to covering the fibers with polymer resins. In addition, aramid fibers, especially the acid spun type, are reported to undergo a notable degradation in concrete. Furthermore, PAN and PET fibers are susceptible to some levels of degradation in alkaline environments. The other reviewed fibers, however, have shown great chemical stability in concrete.

When FRC is subjected to external loads, fibers tend to fail by pull-out and/or rupture. The occurrence of these two modes of failure is a function of fiber’s elastic modulus and fiber-matrix bond. As the modulus of elasticity decreases and the fiber-matrix bond increases, the majority of fibers tend to fail due to rupture. The bond between the individual fibers and the concrete matrix is often governed by the concrete’s properties, such as water-to-cement ratio, and fiber characteristics, such as material, length, and shape. One of the main reasons for the addition of fibers to a concrete mixture is their ability to enhance the concrete’s post-peak mechanical properties, in terms of ductility and residual strength. Thus, a high fiber-matrix bond that leads to a sudden fiber rupture is not favorable. On the other hand, a weak fiber-matrix bond introduces other issues, particularly with limiting the capability of fibers to bridge the cracks. Therefore, an optimum bond is desired for fibers to have the best efficiency. PVA fibers form a hydrogenic bond with the concrete matrix, while the other fibers primarily have a mechanical bond. Thus, PVA fibers tend to fail due to rupture and a high fiber-matrix bond is expected from them. Nylon, PAN, and PO fibers are reported to make a relatively strong bond with the concrete, due to their swelling and increase of friction, irregular cross-section, and damage during the mixing process, respectively. In contrary, PE fibers form a relatively weak bond with the concrete matrix, presenting a challenge to its use in FRC. Several methods have been attempted, with various degrees of success, to increase the fiber-matrix bond. Among the examples are using the fibrillated form, changing the shape of individual fibers, and adding coating materials.

### 8.2. Workability

Regardless of the dosage and characteristics of fibers, the addition of fibers decreases the workability of concrete. This is further exacerbated by increasing the fiber content. Longer fibers can cause a higher friction with fresh concrete, further reducing the workability of FRC mixtures. However, it should be noted that, in a fixed volume fraction, shorter fibers cause a higher decrease in workability, due to their higher surface areas. Studies have shown that using proper admixtures, such as water reducers and pozzolans, in addition to employing appropriate mixing equipment, increase the maximum amount of fibers that can be included in the FRC without causing workability issues. PVA fibers can significantly decrease the workability of FRC due to their water absorption characteristics. Nylon fibers are also reported to have some water absorption. While this helps them further disperse in the concrete at low volume fractions, a significant reduction in the workability is observed at high volume fractions. Compared to other fiber types, basalt glass fibers are reported to mix well with concrete, mainly due to having a density similar to the concrete’s density.

### 8.3. Pre-Peak Mechanical Properties

The studies available in the literature have reported sometimes contrary observations regarding the effects of fiber addition on the compressive strength of FRC. This can be attributed to different properties of concrete and fibers used in the experiments. When it comes to compressive strength, dense packing plays a key role to ensure that the entrapped air is minimum. Therefore, regardless of fiber type, using short fibers with a low dosage can lead to an increase in the compressive strength. This can be achieved well, especially in the concrete mixtures made with a high water-to-cement ratio. On the other hand, fibers have been consistently reported as a proper addition to improve the concrete’s splitting tensile and flexural strengths, where macrofibers have led to more pronounced improvements in comparison to microfibers. Increasing the fiber content can result in improved splitting tensile and flexural strengths, as long as fibers are well dispersed. In general, the higher the fiber’s modulus of elasticity, the better it can enhance the splitting tensile and flexural strengths of the concrete. Comparative studies have shown that glass fibers can improve the flexural strength of concrete more than PO fibers, while they are both more efficient than high-tenacity PP and nylon fibers. High-modulus carbon and aramid fibers also greatly help with augmenting the splitting tensile and flexural strengths of the concrete, as long as the required fiber-matrix bond is achieved.

### 8.4. Post-Peak Mechanical Properties

One of the main limitations of the plain concrete is its brittle behavior after reaching the ultimate strength, which is captured as a peak and then a sharp drop in the stress-strain curve. In order to overcome this drawback, fibers are incorporated into the mixture to enhance the concrete’s post-peak mechanical properties, which span ductility, toughness, and residual strength. Even low volumes of fibers have proven effective in improving all of the post-peak mechanical properties of the concrete, except for some reported cases regarding the compressive energy absorption. The reason for the overall positive contribution of fibers is their ability to bridge over cracks, facilitating the transfer of loads from one end to the other end of the crack. Therefore, higher fiber contents provide more bridging pathways, which help with conveying more stresses, thus, further increasing the post-peak mechanical properties of FRC. The past studies have shown that microfibers are more effective in controlling microcracks, whereas macrofibers deliver a better performance in limiting macrocracks. Therefore, macrofibers can be more efficient in enhancing the post-peak properties of the concrete since macrocracks are generated after the peak of the stress-strain curve is reached. High-modulus fibers, such as carbon and aramid, followed by glass fibers, have shown a great potential to improve the post-peak mechanical properties of FRC. On the other hand, the contribution of nylon fibers to the post-peak mechanical properties is expected to be the least compared to PP, PVA, and HSPE fibers.

### 8.5. Shrinkage

Since concrete has a low tensile strength, shrinkage-induced cracks caused due to water consumption and/or loss can be a great concern for long-term durability. This is because when the concrete loses water as a result of excessive evaporation, internal tensile stresses are generated with the possibility of exceeding the concrete’s tensile strength, leading to the formation and propagation of cracks. To address the issue of shrinkage-induced cracks, adding fibers to the concrete has been investigated in several studies. Regardless of their material characteristics, fibers can reduce the crack width and number of cracks, while delaying the time of the first crack. Additionally, it has been found that the higher the fiber content, the lower the shrinkage-induced cracks to the extent that such cracks can be entirely eliminated. The fiber aspect ratio is one of the important factors, affecting the performance of FRC subjected to shrinkage. In general, fibers with a high aspect ratio have a better performance than those with a low aspect ratio. This can be confirmed with the great potential of fibrillated PP fibers to limit shrinkage-induced cracks.

### 8.6. Extreme Temperature Resistance

When concrete is exposed to extreme temperatures or fire, the water inside the concrete matrix evaporates, generating an inside vapor pressure in the form of tensile stresses that can lead to the explosion and spalling of concrete. However, the addition of fibers is proven to be an efficient way to reinforce the concrete against extreme temperatures. Fibers enable FRC to withstand high temperatures with two methods: firstly, fibers with a high melting temperature (e.g., carbon, aramid, and glass fibers) can increase the tensile strength of concrete. Therefore, they can delay the explosion time and help concrete retain a high residual strength after the fire. Secondly, fibers with a low melting point (e.g., PP and PE fibers) melt during the fire, forming internal channels that serve as evacuation pathways for water vapors. If a proper combination of fiber dosage and length is employed, the concrete’s explosion and spalling can be minimized or even eliminated. The available studies suggest that the FRC products with the fibers that fall into the second category have a better performance overall, when exposed to elongated extreme temperatures.

## 9. Conclusions

In this review study, a variety of fibers have been investigated for their contribution to different fresh and hardened properties of concrete. Additionally, they have been compared with each other in order to signify their potential in altering each of the properties of interest. From this holistic investigation, the capabilities and limitations of each fiber type can be summarized as:

- PP fibers are one of the most cost-effective concrete fibers. This advantage, paired with an excellent chemical stability in the concrete environment, satisfactory mechanical properties, and wide availability, has made the PP fibers one of the popular concrete fibers. The fibrillated PP microfibers are primarily used for plastic shrinkage crack control, while the monofilament PP macrofibers are employed for controlling the cracks caused by external loads, temperature gradients, or drying shrinkage.
- Nylon fibers can absorb the mixing water, and in turn, reduce the workability more than other concrete fibers. These aspects limit the application of nylon fibers to relatively low fiber volumes, especially if microfibers are used. Another limitation of nylon fibers is that, while they provide advantages similar to PP fibers in concrete, they are, in general, more expensive. Increasing attention to recycled nylon fibers can help decrease the unit cost of nylon fibers with the possible use for thermal and plastic shrinkage crack control purposes.
- PVA fibers form a strong chemical bond with the concrete matrix, increasing the possibility of fiber rupture under external loads, which is not a favorable feature where an increase in post-peak mechanical properties is needed. This feature, along with the relatively high cost of PVA fibers and their significant water absorption, which decreases the mixture’s workability more than other fibers, can limit the application of PVA fibers in FRC products. Thus, they are not as readily available as other less expensive synthetic fibers.
- PO fibers have a relatively low elastic modulus, similar to PP fibers, causing a relatively low residual strength at small crack widths. Most concrete fiber suppliers provide some forms of PO fibers, as they work well for crack controlling purposes. PO fibers are relatively inexpensive and fall in the same price range as PP fibers, making them one of the least expensive concrete fibers. However, they are not well represented in the literature, most likely due to the absence of a widespread need to them in practice because of the popularity and abundance of PP microfibers.
- Carbon fibers can withstand the alkaline environment of concrete better than glass fibers. They also have a (relatively) high strength-to-weight ratio. However, due to the issues observed during the mixing process of carbon macrofibers with conventional methods, they are not commonly used, especially in the mixtures that contain coarse aggregates. Further to the high price of most carbon fibers, they are often less effective than other synthetic fibers for several concrete applications. Thus, carbon fibers are considered an expensive specialty fiber in the concrete industry.
- As a result of low strength and stiffness, the FRC products made with conventional PE fibers can suffer from poor mechanical properties. However, high-strength and high-stiffness PE fibers have shown satisfactory mechanical properties with a potential to be used in cementitious composites. However, HSPE fibers are not very practical in conventional concrete applications, mainly because of their (relatively) weak bond with concrete.
- The use of PET fibers in FRC has been limited to laboratory tests and research investigations at the time of this review. It is expected that, as the production technology and product quality of recycled PET fibers improve in the future, these fibers gain traction in the concrete industry, owing to their economic and environmental benefits over traditional synthetic fibers.
- In the category of acrylic fibers, PAN microfibers have been found to offer effective solutions, as they can provide benefits similar to other low-strength/modulus fibers. However, compared to other common synthetic fibers, the literature suggests that acrylic fibers more adversely affect the workability of the concrete mixture, while they can provide an increased fiber-matrix bond strength, along with a significant residual strength at small crack openings. The limited general use of PAN fibers in the concrete mixtures that contain coarse aggregates is likely because other less expensive synthetic fibers can provide similar benefits, especially in the absence of acrylic macrofiber production.
- The main drawback of aramid fibers for FRC applications is their cost. Since aramid fibers are relatively expensive and may not provide enough additional benefits over other common concrete fibers, their use has been limited, which can justify the limited number of relevant studies available in the literature. However, recent works on Kevlar and Technora fibers have shown promising reinforcing potential, which can be further utilized, especially if the cost drops.
- AR silica glass fibers are able to significantly improve the strength and ductility of concrete, owing to their relatively high strength and stiffness. There are inconsistencies in the workability reported for the FRC mixtures made with AR silica glass fibers, but this can be attributed to the fact that the fibers can come in a wide range of sizes and surface areas. Degradation of silica glass fibers in concrete is often a concern, which can be addressed with an adequate zirconia content, proper sizing application, concrete binder adjustment, or even polymer impregnation. The AR silica glass fibers are effectively used as concrete fibers in various applications with a wide availability and relatively low price.
- Basalt glass fibers have shown degradation issues in the concrete’s alkaline environment. Similar to silica glass fibers, various actions have been taken to increase their long-term stability in concrete. Basalt microfibers are known to be effective for increasing the splitting tensile and flexural strengths of FRC, further to reducing the plastic shrinkage cracks. BFRP macrofibers have shown a great potential as an effective solution to improve the post-crack performance of FRC and control the propagation of cracks. The basalt glass fibers are anticipated to gain popularity in the concrete industry, as the production increases and new applications are identified.

FRC products are becoming more popular in the concrete industry and are anticipated to continue to grow as researchers, engineers, and contractors become more familiar with designing, mixing, and placing FRC, while the new codes and standards for structural concrete accept the strength and service life benefits that can be gained from adding randomly dispersed discrete fibers to the concrete. Holistic investigations, similar to the investigation presented in the current review study, are expected to pave the way to make informed decisions regarding the fiber types of choice, depending on not only their cost and availability, but also the fresh and hardened properties of FRC desired for a wide variety of concrete applications.

## Figures and Tables

**Table 1:** Select properties of reviewed polymer fibers.

Property	Polymer Fibers								
	Polypropylene	Nylon	Polyvinyl Alcohol	Polyolefin	Carbon	Polyethylene	Polyester	Acrylic	Aramid
Tensile Strength (MPa)	60–700	300–950	850–1600	300–700	1500–7000	40–3000	250–1000	300–1000	2300–3400
Elastic Modulus (GPa)	1.5–10.0	3.0–5.4	25–41	3.0–10	30–500	0.5–120	10–20	3.8–17	70–143
Ultimate Strain (%)	8.0–15.0	10.0–20.0	5.0–7.0	5.0–15.0	0.5–2.5	3.0–80.0	10.0–50.0	7.5–50.0	2.0–4.5
Water Absorption (%)	0	2.5–5.0	0.1–1.0	0	0	0	0.2–0.6	1.0–2.5	1.2–4.0
Specific Gravity	0.90–0.95	1.13–1.15	1.30	0.90–0.95	1.60–1.90	0.92–0.98	1.32–1.38	0.91–1.20	1.39–1.47

**Table 2:** Select properties of reviewed glass fibers.

Property	Glass Fibers		
	Silica Glass	Basalt Glass	GFRP/BFRP
Tensile Strength (MPa)	1700–4600	1800–4800	1080
Elastic Modulus (GPa)	72–89	72–110	44
Ultimate Strain (%)	2.0–3.5	2.0–3.5	2.0–3.0
Water Absorption (%)	0	0	0
Specific Gravity	2.6–2.7	2.55–2.8	1.9–2.1

**Table 3:** A summary assessment of the main characteristics of polymer and glass fibers in FRC.

Fiber	Stability	Bond	Workability	Pre-Peak Mechanical Properties		Post-Peak Mechanical Properties	Shrinkage	Temperature and Fire Resistance		Cost	Availability
				Compressive Strength	Tensile & Flexural Strength			Melting Point	Performance		
PP	E	F–G	G	W–F	F	F	G–E	Low	F–G	E	E
Nylon	E	G	F–G	W–F	F	W–F	W	Low	E	F–G	G
PVA	E	E	W	W–F	F–G	F	F–G	Low	G	W	F
PO	E	G	G	W–F	F–G	F	G	Low	NI	G–E	G
Carbon	E	F–G	G	F–G	G–E	G–E	G	High	F–G	W	W
HSPE	E	W	G	W–F	F	F	G–E	Low	NI	W	NI
PET	F–G	F–G	G	W–F	F	F	F	Low	F–G	NI	NI
PAN	F–G	G	G	W–F	F	F	NI	Low	NI	W	F–G
Aramid	W–G	F	G	W–F	G–E	G–E	NI	High	F–G	W	W
Silica Glass	W	G	G	W–G	G	F–G	F	High	F	G	E
Basalt Glass	W	G	E	W–G	E	G–E	F–G	Very High	E	G–E	F

W: weak, F: fair, G: good, E: excellent, and NI: not enough information.
